# Genetic and phenotypic stability of poliovirus shed from infants who received novel type 2 or Sabin type 2 oral poliovirus vaccines in Panama: an analysis of two clinical trials

**DOI:** 10.1016/S2666-5247(22)00254-3

**Published:** 2022-12

**Authors:** Rahnuma Wahid, Laina D Mercer, Tirza De Leon, Rodrigo DeAntonio, Xavier Sáez-Llorens, Andrew Macadam, Konstantin Chumakov, Jeroen Strating, Björn Koel, Jennifer L Konopka-Anstadt, M Steven Oberste, Cara C Burns, Raul Andino, Erman Tritama, Ananda S Bandyopadhyay, Gabriela Aguirre, Ricardo Rüttimann, Chris Gast, John O Konz

**Affiliations:** a Center for Vaccine Innovation and Access, PATH, Seattle, WA, USA; b Hospital Materno Infantil José Domingo De Obaldía, David, Panama; c CEVAXIN, Centro de Vacunación e Investigación, Panama City, Panama; d Department of Infectious Diseases, Hospital del Niño Dr José Renán Esquivel and Sistema Nacional de Investigación at Secretaria Nacional de Ciencia y Tecnologia, Panama City, Panama; e Division of Virology, National Institute for Biological Standards and Control, South Mimms, UK; f Center for Biologics Evaluation and Research, Food and Drug Administration, Silver Spring, MD, USA; g Global Virus Network Center of Excellence, Baltimore, MD, USA; h Viroclinics Xplore, Viroclinics Biosciences, Rotterdam, Netherlands; i Viroclinics Biosciences, Rotterdam, Netherlands; j Division of Viral Diseases, Centers for Disease Control and Prevention, Atlanta, GA, USA; k Department of Microbiology and Immunology, University of California, San Francisco, CA, USA; l Research and Development Division, PT Bio Farma, Bandung, West Java, Indonesia; m Bill & Melinda Gates Foundation, Seattle, WA, USA; n Fighting Infectious Diseases in Emerging Countries, Miami, FL, USA

## Abstract

**Background:**

Sabin strains used in oral poliovirus vaccines (OPV) can revert to virulence and, in rare instances, cause disease or generate vaccine-derived strains leading to outbreaks in areas of low immunisation coverage. A novel OPV2 (nOPV2) was designed to stabilise the viral genome against reversion and reduce recombination events that might lead to virulent strains. In this study, we evaluated the genetic and phenotypic stability of shed poliovirus following administration of one dose of monovalent OPV2 (mOPV2) or nOPV2 to infants aged 18–22 weeks.

**Methods:**

In two similarly designed clinical trials (NCT02521974 and NCT03554798) conducted in Panama, infants aged 18–22-weeks, after immunisation with three doses of bivalent OPV (types 1 and 3) and one dose of inactivated poliovirus vaccine, were administered one or two doses of mOPV2 or nOPV2. In this analysis of two clinical trials, faecally shed polioviruses following one dose of mOPV2 or nOPV2 were isolated from stools meeting predetermined criteria related to sample timing and viral presence and quantity and assessed for nucleotide polymorphisms using next-generation sequencing. A transgenic mouse neurovirulence test was adapted to assess the effect of the possible phenotypic reversion of shed mOPV2 and nOPV2 with a logistic regression model.

**Findings:**

Of the 91 eligible samples, 86 were able to be sequenced, with 72 evaluated in the transgenic mouse assay. Sabin-2 poliovirus reverts rapidly at nucleotide 481, the primary attenuation site in domain V of the 5ʹ untranslated region of the genome. There was no evidence of neurovirulence-increasing polymorphisms in domain V of shed nOPV2. Reversion of shed Sabin-2 virus corresponded with unadjusted paralysis rates of 47·6% at the 4 log_10_ 50% cell culture infectious dose (CCID_50_) and 76·7% at the 5 log_10_ CCID_50_ inoculum levels, with rates of 2·8% for 4 log_10_ CCID_50_ and 11·8% for 5 log_10_ CCID_50_ observed for shed nOPV2 samples. The estimated adjusted odds ratio at 4·5 log_10_ of 0·007 (95% CI 0·002–0·023; p<0·0001) indicates significantly reduced odds of mouse paralysis from virus obtained from nOPV2 recipients compared with mOPV2 recipients.

**Interpretation:**

The data indicate increased genetic stability of domain V of nOPV2 relative to mOPV2, with significantly lower neurovirulence of shed nOPV2 virus compared with shed mOPV2. While this vaccine is currently being deployed under an emergency use listing, the data on the genetic stability of nOPV2 will support further regulatory and policy decision-making regarding use of nOPV2 in outbreak responses.

**Funding:**

Bill & Melinda Gates Foundation.

## Introduction

Vaccination with Sabin oral poliovirus vaccine (OPV) results in robust intestinal and humoral immunity, which are key to the control of poliomyelitis, but the inherent genetic instability of the OPV strains is well documented.^1^, ^2^, ^3^ During replication, the OPV strains can lose their attenuating mutations in domain V of the 5ʹ untranslated region, which results in reacquisition of virulence.^1^, ^4^, ^5^ In rare cases, the reverted viruses can cause vaccine-associated paralytic polio (VAPP) in recipients or their immediate contacts. After prolonged circulation in under-immunised populations, reverted viruses can become highly transmissible circulating vaccine-derived poliovirus (cVDPV).

With the certification of the global eradication of wild type 2 poliovirus the routine use of the type 2 component of Sabin OPV was ceased globally in 2016. Although this switch has been successful in most regions, waning immunity to type 2 viruses in areas of low vaccine coverage has contributed to increasing numbers of outbreaks of type 2 cVDPV, with consequent increases in cases of paralysis.^6^ In some cases, use of monovalent OPV2 (mOPV2) to combat outbreaks is seeding outbreaks in adjacent areas.^7^, ^8^, ^9^

**Research in context**

**Evidence before this study**

The widespread transmission of type 2 vaccine-derived poliovirus constitutes a Public Health Emergency of International Concern. A novel type 2 oral polio vaccine (nOPV2) received a WHO emergency use listing in November, 2020, to respond to this crisis based on promising clinical data. We previously reported in two publications in *The Lancet* four clinical studies that demonstrated the safety, tolerability, and immunogenicity of nOPV2 in adults, young children, and infants compared with monovalent Sabin OPV2 (mOPV2). The key driver of a change from mOPV2 to use of the novel vaccine in outbreak settings is its improved genetic and phenotypic stability, which has been described in two published reports in *NPJ Vaccines* from studies using shed stool samples obtained from adults and children administered mOPV2 or nOPV2. We did not conduct a literature search as the few published reports regarding novel OPV2 have been generated from the novel OPV2 consortium.

**Added value of this study**

This report represents the first rigorous comparison of genetic and phenotypic stability, conducted with samples from infants, a primary target population of nOPV2 use in outbreak settings, completing the assessment of nOPV2 in the previously reported clinical studies. We show that the vaccine viruses shed by infants administered nOPV2 have significantly reduced reversion to virulence compared with polioviruses shed by infants administered mOPV2. We also show the improved stability of the primary attenuation site, domain V, of nOPV2 compared with mOPV2.

**Implications of all the available evidence**

The data from this key study population provide a crucial demonstration of the better genetic and phenotypic stability of shed nOPV2 strains compared with shed mOPV2 in infants. These data suggest that nOPV2 should be associated with less paralytic disease and potentially a lower risk of seeding new outbreaks, the objective of the development of these new vaccines. While this vaccine is currently being deployed under an emergency use listing, the data on the genetic stability of nOPV2 will support further regulatory and policy decision-making regarding use of nOPV2 in outbreak responses.

Novel OPV2 (nOPV2) was designed to provide similar protection as mOPV2 but with reduced risk of loss of attenuation.^10^, ^11^, ^12^ The safety, immunogenicity, viral shedding, and genetic stability results from phase 1 and 2 studies of nOPV2 in adults, children, and infants have been reported.^13^, ^14^

Our study supplements previous reports from similarly designed phase 4 and phase 2 studies^14^ conducted in Panama, where according to the WHO/UNICEF Estimates of National Immunization Coverage data (July 7, 2022) national coverage of a third dose of OPV was estimated to increase from 72% in 2015 to 86% in 2016 while the control phase 4 study was conducted and was estimated to be 88% in 2018 and 2019 while the phase 2 study was being conducted. In this Article, we used established methods^15^ to evaluate the genetic and phenotypic stability of OPV2 strains following administration of one dose of mOPV2 or nOPV2 to infants aged 18 to 22 weeks. We compared the neurovirulence potential of shed nOPV2 with shed mOPV2 in a modified transgenic mouse neurovirulence test (mTgmNVT) and assessed genetic heterogeneity of the shed virus using next-generation sequencing (NGS).

## Methods

### Study design and participants

This study is an analysis of shed virus in samples obtained from two trials. The first is a phase 4 trial (M2; NCT02521974) conducted in 2015–16 before the global switch to bivalent OPV (bOPV) containing only types 1 and 3, in which mOPV2 was administered to 110 healthy infants age 18–22 weeks who received three doses of bOPV and one dose of inactivated polio vaccine at least 4 weeks before administration of mOPV2. The second is a phase 2 trial (M5; NCT03554798) in 2018–19, in which 150 healthy infants age 18–22 weeks received at least one oral dose of nOPV2 at least 4 weeks after receiving three doses of bOPV and one dose of inactivated polio vaccine.^14^ Note that the M5 trial included double-blinded cohorts receiving one of two candidate nOPV2 vaccines; however, genetic and phenotypic evaluation on shed virus was limited to a single candidate strain (nOPV2 candidate 1) for these cohorts. Full eligibility criteria is described in the report of the clinical trials.^14^ Both studies were performed in the Cevaxin Vaccination Network, Panama City, Panama. Ethical approval was received from the ethical review committee of the Hospital del Niño Dr José Renán Esquivel (Panama City, Panama) and written informed consent was obtained from parents or legal guardian(s) at enrolment.

### Procedures

Polio Sabin Mono Two (mOPV2), a type 2 Sabin strain, was manufactured by GlaxoSmithKline Biologicals, Rixensart, Belgium. A dose consisted of 2 drops (0·1 mL) containing no less than 10^5·0^ 50% cell culture infectious dose (CCID_50_) virus.

nOPV2, referred to as nOPV2 candidate 1 (S2/cre5/S15domV/Rec1/HiFi3) in earlier publications,^12^ was manufactured by PT Bio Farma, Bandung, Indonesia. For the analyses described in this Article, infants received a nOPV2 dose of approximately 10^6·0^ CCID_50_ in 1 mL.

In each study, stool samples were collected daily following each vaccination until day 10, and then on days 14, 21, and 28, and were stored at −20°C and shipped periodically for analysis to the Centers for Disease Control and Prevention (CDC; Atlanta, GA, USA) as previously described.^15^ Stools were assessed for presence of poliovirus types 1, 2, and 3. Stools positive for type 2 poliovirus only were titrated and evaluated in this study. For each participant, the last sample collected following dose 1 with at least 4·00 log_10_ CCID_50_ type 2 virus per g of stool was termed the exploratory endpoint specimen (EES; appendix pp 2–3).^15^ Stool suspensions (10% w/v in Eagle's minimum essential medium, Gibco) of the EES were prepared for NGS. To achieve the required virus concentration for mouse neurovirulence assays, virus was amplified in HEp-2C cells (ATCC, CCL-23). EES stool suspensions and virus isolates were assigned unique identifiers and shipped on dry ice to Viroclinics Biosciences (Schaijk, Netherlands) and stored frozen at −80°C until analysis. Presence and quantitation of virus were assessed independently for each study, but M2 and M5 EES were tested concomitantly in NGS and mouse neurovirulence assays, with analysts blinded to test samples in the neurovirulence tests. The experiments using transgenic mice expressing the human poliovirus receptor (Tg-PVR21 mice), were conducted in accordance with Dutch and European animal ethical standards.

### Neurovirulence of shed virus

The virus titrations for EES and controls, and the mTgmNVT were described previously.^15^, ^16^ Briefly, for each EES, ten Tg-PVR21 mice aged 6–8 weeks (CLEA, Tokyo, Japan; five male and five female) were randomly assigned to receive intraspinal inoculations with 5 μL of 4 log_10_ or 5 log_10_ CCID_50_ amplified virus from EES or control virus. Virus from EES were amplified in a single round of infection of Hep2C cells under conditions previously shown to be non-selective. These doses were selected for the assay to be sensitive to clinically relevant Sabin-2 reversion. The controls were 20 mice inoculated with Sabin-2 (SO + 2/II) at 6 log_10_ CCID_50_ doses and ten mice inoculated with SO + 2/II 5 log_10_ CCID_50_ doses. A Sabin-2 molecular construct with reversion at two genome sites (481a→g and 2908a→g) was inoculated into 20 mice at 4 log_10_ CCID_50_ dose level as a positive control. Back-titrations of the diluted samples confirmed that inoculum titres were within 0·5 log_10_ CCID_50_ of the nominal dose. Inoculated mice were monitored for paresis or paralysis over a 14-day period and assigned a clinical score per established protocol.^15^, ^17^

For nOPV2 samples that induced 40% or more paralysis, the paralytic dose for 50% of mice (PD_50_) was determined. For these tests, groups of ten Tg-PVR21 mice were inoculated with five different dose levels of each sample.

### NGS of shed virus

NGS and data analysis were performed on viral RNA isolated from both cell-culture-amplified virus and from 10% stool suspensions of the EES of each participant, using previously described methods.^15^, ^18^, ^19^ In summary, viral RNA was isolated and cDNA prepared by full-length reverse transcription of the genome. Tagmentation and library preparation used Illumina DNA Prep (M) Tagmentation (Illumina, San Diego, CA, USA), followed by 300-cycle paired-end sequencing using Miseq reagent kit v3 on a Miseq instrument (Illumina, San Diego, CA, USA). Reads were mapped against nOPV2 or Sabin 2 reference sequence (Genbank accession number AY184220) using a custom bioinformatics pipeline.

### Statistical analysis

A binomial logistic generalised linear regression mixed model with mouse sex as a factor and subject (EES)-specific random effects was fitted with SAS (version 9.4) to mouse paralysis data for each vaccine group separately, yielding estimates of paralysis rate. For comparison of paralysis rate of shed nOPV2 versus shed mOPV2, two additional similar models were fitted that also included a term to estimate the odds ratio of paralysis for the virus shed from nOPV2 recipients relative to mOPV2 recipients. This adjusted odds ratio (aOR) is adjusted for mouse sex imbalance owing to mice excluded from evaluation due to reasons unrelated to the inoculum. The primary inferential comparison between groups was predefined to be based on the model-estimated aOR (nOPV2/mOPV2 ratio) at 4·5 log_10_ CCID_50_ (midpoint of inocula) and its corresponding confidence interval. We used inverse probability weighting to account for stratified subsampling of M5 EES according to post-vaccination week, as described in the appendix (pp 2–4). Estimated PD_50_ values, averaged across mouse sex, were obtained by inverting the regression models. As an exploratory analysis, a similar model was fitted in R (version 4.0.0) to mouse paralysis data for each vaccine group and dose level to assess the relationship between frequency of mutation at key sites and mouse paralysis. See the appendix (pp 2–7) for a detailed description of the statistical methods.

### Role of the funding source

The funder of the study had no role in data collection, data analysis, or data interpretation. ASB was an employee of the study funder and was involved in the clinical trial design and writing of the report.

## Results

Although the global cessation of OPV2 usage in routine immunisation required that the M2 and M5 studies were conducted non-contemporaneously, given the similar vaccination histories of the participants in both cohorts, and overall similar rates of viral shedding among nOPV2 and mOPV2 recipients early after vaccination,^20^ similar numbers of EES were anticipated from these study groups. 36 EES were identified from 110 participants in the M2 study compared with 55 EES for nOPV2 from a cohort of 150 participants in the M5 study.

**Figure 1:**
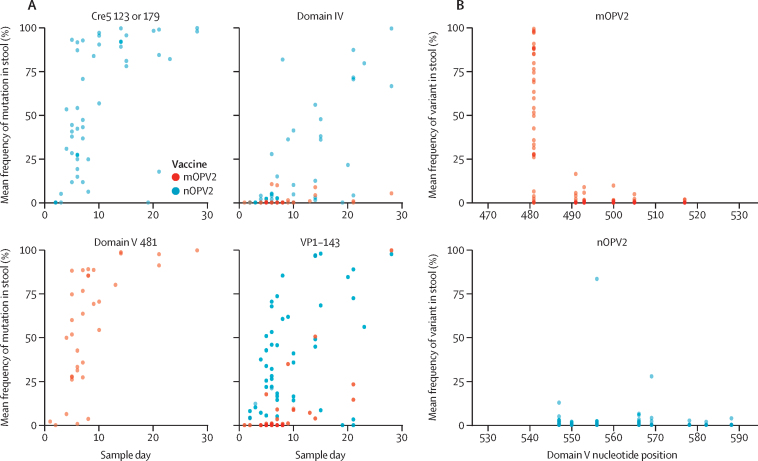
Frequency of mutations in EES for mOPV2 and nOPV2 (A) Frequency of key mutations in EES as a function of day following vaccine administration. Mean frequencies of SNPs from two stool replicates (if present) for each EES from each trial M2 (mOPV2) and M5 (nOPV2) are plotted. If an SNP is only present in one stool that value is presented. If an SNP is not present in either stool, the SNP is assigned 0 for plotting purposes. Mutations at cre5 nucleotides 123 and 179 are presented in aggregate for nOPV2. Mutations in domain IV (nucleotide 398 for mOPV2 or nucleotide 459 for nOPV2), domain V (nucleotide 481 for mOPV2), and VP1-143 (nucleotides 2908–2910 for mOPV2 or nucleotides 2969–2971 for nOPV2) are presented in aggregate as a function of EES day. All reported SNPs have Q scores of 30 or more. (B) Frequency of domain V polymorphisms (nucleotides 468–535 for mOPV2 or nucleotides 529–596 for nOPV2) at each nucleotide position from 34 EES of shed mOPV2 and 52 EES of shed nOPV2. Mean frequencies of SNPs from two stool replicates (if present) for each EES are plotted. If SNP is only present in one stool the other stool is assumed to be 0%. SNPs shown meet the reporting criteria described in the Methods. All reported SNPs have Q scores of 30 or more. Cre5=cis-acting replication element 5. EES=exploratory endpoint specimen. mOPV2=monovalent type 2 oral poliovirus vaccine. nOPV2=novel type 2 oral poliovirus vaccine. SNP=single-nucleotide polymorphism.

NGS was performed on shed virus (two replicates of 10% stool suspension per EES) from 36 EES from the M2 study ranging from 1 day to 28 days after mOPV2 administration. 34 EES were successfully sequenced, with two excluded due to insufficient DNA concentration following NGS library preparation. Data from the key genome sites are shown in figure 1A. The 398u→c single-nucleotide polymorphism (SNP) in domain IV of the 5ʹ untranslated region, known to be related to virus fitness and adaptation, was observed at low frequency in several EES. Mutations in VP1-143, the secondary attenuation site, were observed at day 4 and later EES (VP1-Ile143Thr fixed [a polymorphism that was present at or near 100% frequency] in day 28 EES). Reversion at position 481, the primary attenuation site, was observed consistently by day 4 EES and 481G was fixed in the day 14 and later EES. Other low-level polymorphisms were observed in domain V (figure 1B).

NGS was performed on 52 EES (three of 55 EES had insufficient DNA for sequencing) ranging from 2 to 28 days following nOPV2 administration. SNPs in the key modified and known attenuating regions are summarised in figure 1 and in the appendix (pp 14–29). Mutations at the base of the stem in the relocated cis-acting replication element (cre5) at nucleotides 123 and 179 were observed in day 4 to 28 EES at variable levels up to 100% frequency. Positions 123 and 179 are opposite each other in a hairpin structure in a u–g pairing. The 123u→c variant results in a c–g pair, and the 179g→a variant results in a u–a pair. Other SNPs were observed in the cre5 region at low levels (appendix pp 17–20); several samples had minority (<10% average frequency in samples where present) accumulations of 121c→u or 181a→g polymorphisms, or both. These polymorphisms allow for an additional base pair at positions 121–181 through formation of a u–a or c–g pair, probably extending the length of the stem in the cre structure.

Variation was also noted at position 459 in domain IV, corresponding to nucleotide 398 in Sabin-2. 459u→c increased in frequency in later EES and is fixed in a day 28 EES. By contrast to mOPV2 EES, mutations which result in reversion of VP1-143 were observed consistently, with several nOPV2 EES at 50% variant frequency or higher (fixed in at day 28 EES). Changes involved in the reversion of VP1-143 are observed at nucleotides 2969 and 2970 and changes at both positions were sometimes detected within the same sample. The 2969 and 2970 variants are reported as SNPs but could represent dinucleotide polymorphisms. Generally, the data suggest these two locations of mutations might undergo more rapid selection for nOPV2 than mOPV2.

No reverting polymorphisms (defined as strengthening of a dinucleotide pairing in the stem regions of domain V) were observed in the novel domain V (nucleotides 468–535) of shed nOPV2 (figure 1B). Low-level SNPs (generally ≤10% in any replicate) were observed in several EES. One SNP, 556c→u, was present at greater than 80% frequency in both stool replicates of a day 28 EES. No other SNPs in domain V were present along with 556c→u in this EES. Two other SNPs were present at greater than 10% frequency (547c→u at approximately 12% in a day 20 EES and 569c→u at approximately 30% in a day 23 EES). None of the observed SNPs are pair forming or strengthening.

To confirm whether the culture-amplified virus was sufficiently representative for neurovirulence assessment, the frequency of mutation in stool samples was compared with the frequency of mutation in the amplified sample for mOPV2 and nOPV2 (appendix p 9). Polymorphism frequencies were reviewed for the attenuation sites and, for nOPV2, SNPs in the relocated cre5. In this comparison, key polymorphisms noted in stool were largely detected in the culture-amplified virus (for use in mTgmNVT) and were generally present at similar levels in the stool and amplified virus. For mOPV2, high concordance was observed for the primary attenuation site polymorphisms (correlation coefficient 0·957) and secondary attenuation site polymorphisms (correlation coefficient 0·811). High concordance for SNPs at nucleotides 123 or 179, 459, and in the codon for VP1-143 was also observed for nOPV2. No evidence of bias from the amplification process was observed. Accordingly, the amplified virus samples were determined to be suitably representative for neurovirulence testing.

To provide a sufficiently robust evaluation of potential neurovirulence of shed virus in mice, preclinical and previous clinical data determined that 36 EES per vaccine (mOPV2 and nOPV2) would provide sufficient power for comparison. No subsampling was therefore necessary for mOPV2 EES, and 36 of the 51 nOPV2 EES (four EES did not produce evaluable virus post culture-amplification) were subsampled with simple random sampling within strata defined by post-vaccination week, focusing on those EES most distal to vaccination (appendix p 30).

**Figure 2:**
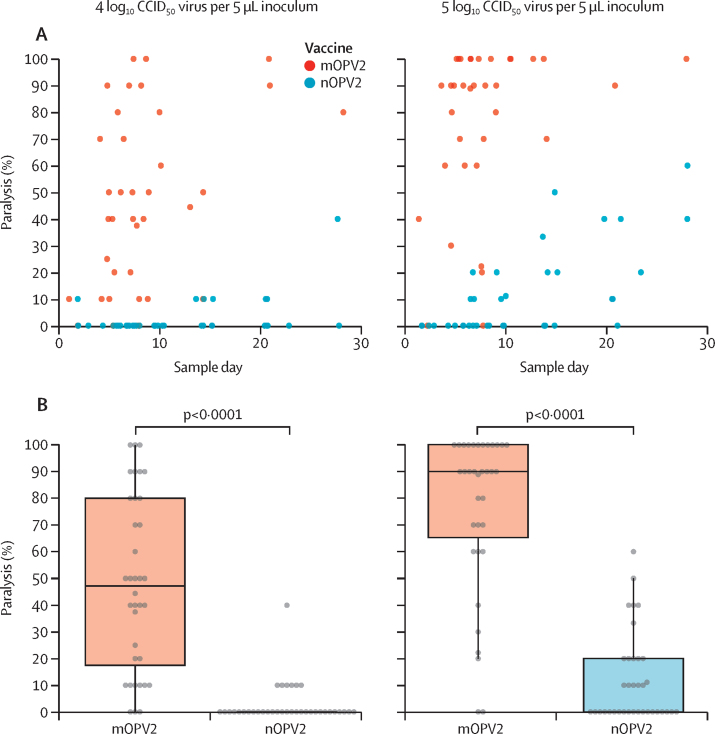
Neurovirulence evaluations of shed virus as a function of collection day (A) and comparatively evaluated between vaccines (B) (A) Measured paralysis rate in modified mouse neurovirulence test. Datapoints are percent paralysis per EES following intraspinal inoculation with 4 log_10_ or 5 log_10_ CCID_50_ virus per 5 μL inoculum per mouse in the mTgmNVT. Paralysis rates for shed virus samples are indicated versus day of EES per vaccine. EES days has been horizontally jittered to improve data visibility. (B) Datapoints are percent paralysis per EES following intraspinal administration of 4 log_10_ or 5 log_10_ CCID_50_ virus inoculum per mouse in the mTgmNVT. The p values indicated are model-based pairwise comparisons of paralysis for shed virus of nOPV2 compared to mOPV2. CCID_50_=50% cell culture infectious dose. EES=exploratory endpoint specimen. mTgmNVT=modified transgenic mouse neurovirulence test. mOPV2=monovalent type 2 oral poliovirus vaccine. nOPV2=novel type 2 oral poliovirus vaccine.

For mOPV2, high paralysis rates were observed at both inoculum levels with EES as early as 4 days after vaccination (figure 2A). The unadjusted proportions paralysed were 47·6% at the 4 log_10_ CCID_50_ dose and 76·7% at the 5 log_10_ CCID_50_ dose (figure 2B). Descriptive model-based analysis estimated the mean paralysis rate at 4·5 log_10_ CCID_50_ of 63·1% (95% CI 46·3–77·2; appendix p 10).

For nOPV2, rates of paralysis increased with later EES days (figure 2A) and a dose-dependent effect was observed (figure 2B). Unadjusted proportions paralysed were 2·8% at the 4 log_10_ CCID_50_ dose and 11·8% at the 5 log_10_ CCID_50_ dose. Descriptive model-based analysis estimated the mean paralysis rate at 4·5 log_10_ CCID_50_ of 0·8% (95% CI 0·2–3·9; appendix p 10). The discrepancy between unadjusted proportions and model-based estimates is due to adjustment for mouse sex and actual inoculum dose, which were generally higher than the targeted 4 log_10_ and 5 log_10_ CCID_50_ doses and is described in the appendix (pp 7, 10). The estimated aOR at 4·5 log_10_ of 0·007 (95% CI 0·002–0.023; p<0·0001) indicates significantly reduced odds of mouse paralysis from virus obtained from nOPV2 recipients compared with mOPV2 recipients.

**Figure 3:**
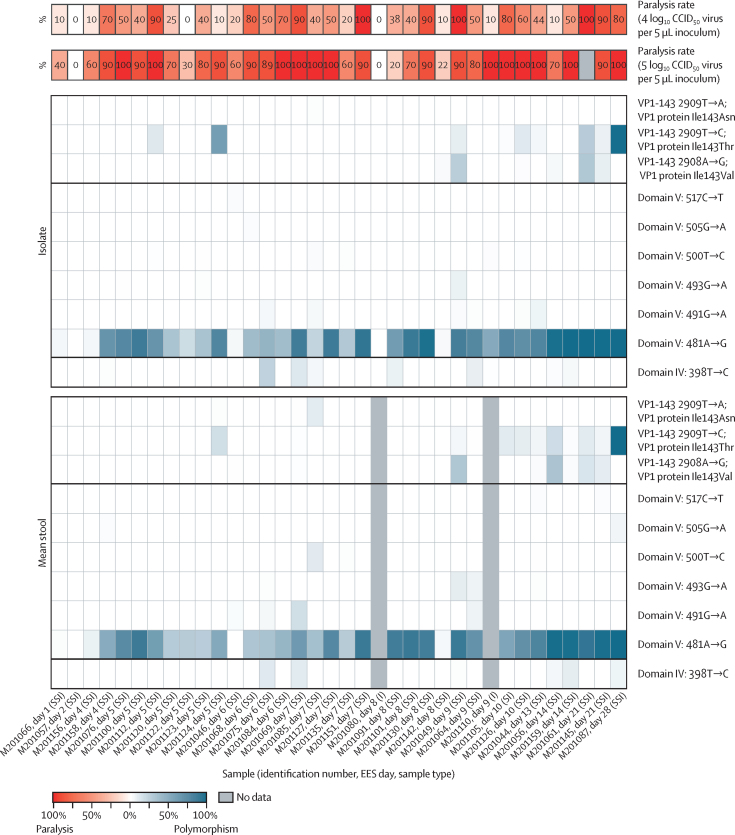
Frequency of polymorphisms at known attenuation sites and mTgmNVT results for each mOPV2 EES EES day shown with mTgmNVT result (red colour gradient) and polymorphism frequency (blue colour gradient) averaged across two stool replicates and culture-amplified virus, if present. Amino acid associated with SNP indicated, if applicable. White cells indicate polymorphism not detected and grey cells indicate mTgmNVT result not available or no NGS data from stool or culture-amplified virus. The NGS pipeline reports SNPs. Coding impact assumes changes are not in common genomes when multiple polymorphisms are observed in VP1-143. All reported SNPs have Q scores of 30 or more. CCID_50_= 50% cell culture infectious dose. EES=exploratory endpoint specimen. I=culture-amplified virus isolate. mTgmNVT=modified transgenic mouse neurovirulence test. mOPV2=monovalent type 2 oral poliovirus vaccine. NGS=next generation sequencing. S=stool replicate. SI=single stool replicate and culture-amplified virus isolate. SNP=single-nucleotide polymorphism. SSI=two stool replicates and culture-amplified virus isolate.

Shed mOPV2 virus reverted rapidly and showed high paralysis rates in the mouse model. NGS data support the high levels of reversion observed for the shed mOPV2 virus (figure 3). Reversion at the main site of attenuation (domain V nucleotide 481) was observed in 34 of 36 EES. These 34 EES showed paralysis in the mTgmNVT whereas the two EES with no detectable 481 reversion did not induce any paralysis.

**Figure 4:**
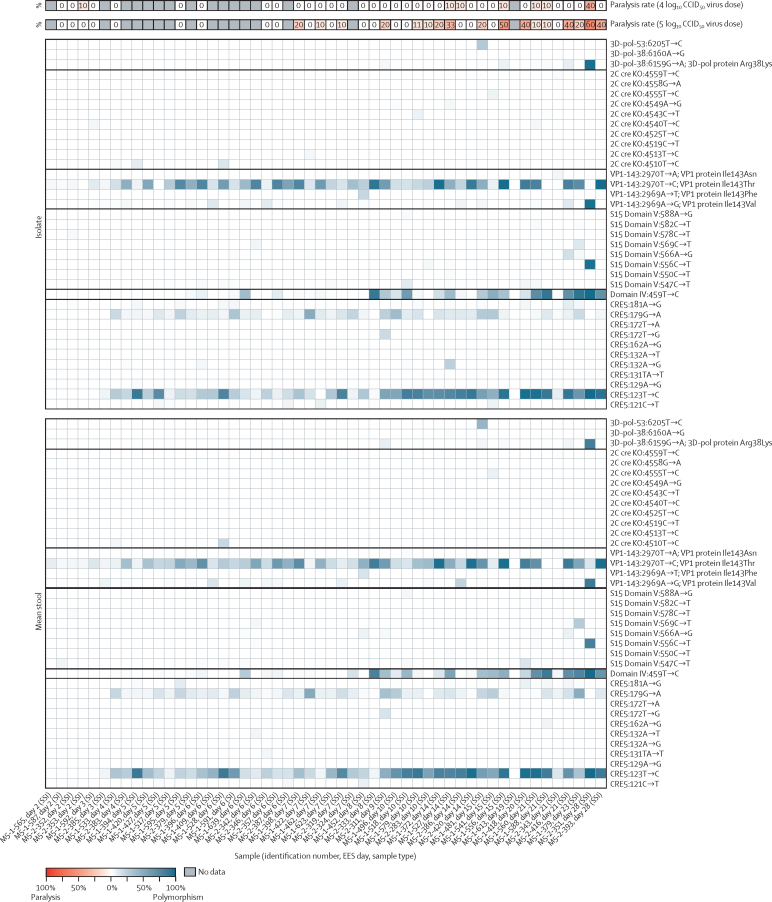
Frequency of polymorphisms at known attenuation sites and mTgmNVT results for each nOPV2 EES EES day shown with mTgmNVT result (red colour gradient) and polymorphism frequency (blue colour gradient) averaged across two stool replicates and culture-amplified virus, if present. Amino acid associated with SNP indicated, if applicable. White cells indicate polymorphism not detected and grey cells indicate mTgmNVT result not available or no NGS data from stool or culture-amplified virus. The NGS pipeline reports SNPs. Coding impact assumes changes are not in common genomes when multiple polymorphisms are observed in VP1-143. All reported SNPs have Q scores of 30 or more. 2C cre KO=2C cre knock out. 3D-pol=3D-polymerase. CCID_50_=50% cell culture infectious dose. Cre5=cis-acting replication element 5. EES=exploratory endpoint specimen. I=culture-amplified virus isolate. mTgmNVT=modified transgenic mouse neurovirulence test. NGS=next generation sequencing. nOPV2=novel oral poliovirus vaccine 2. S=stool replicate. SI=single stool replicate and culture-amplified virus isolate. SNP=single-nucleotide polymorphism. SSI=two stool replicates and culture-amplified virus isolate.

For nOPV2 (figure 4), six of 36 EES showed a single mouse paralysed per sample at the 4 log_10_ dose and 17 of 36 showed 10% or more paralysis at the 5 log_10_ dose. A single day 28 EES (M5-2-351) showed 40% paralysis at the 4 log_10_ CCID_50_ dose and 60% paralysis at the 5 log_10_ CCID_50_ dose. This day 28 EES had fixed mutations at nucleotide 123 in cre5, VP1-143 and at nucleotide 459 with additional fixed changes at nucleotide 556 within domain V and in the 3D polymerase (Arg38Lys, reverting a modification to reduce recombination to the parental amino acid sequence).

nOPV2 EES with higher neurovirulence were evaluated in multi-dose neurovirulence tests to determine the PD_50_ (table 1; appendix p 11). In general, the more virulent samples (lower PD_50_) contain higher levels of 459u→c in domain IV (398u of Sabin-2) and VP1-143 substitutions. Virus from the day 28 EES with fixed mutations at key sites shows the lowest (more virulent) PD_50_ estimate of 5·3 log CCID_50_. The PD_50_ of the day 28 EES aligns with data from a series of virus strains constructed to incorporate key polymorphisms in the nOPV2 background and evaluate their impact on neurovirulence (appendix p 31) and is slightly lower than Sabin-2 vaccine virus (PD_50_ 6·3 log), but notably higher than the observed PD_50_ of 3·5 log for a partially-reverted day 7 shed Sabin-2 virus.^15^ Additionally, 15 of 25 EES collected from mOPV2 recipients 6 days or later after vaccination (figure 3) show 50% or more paralysis at 4 log_10_ CCID_50_ dose, suggesting that PD_50_ would generally be less than 4 log for these Sabin-2 viruses.

**Table 1:** PD50 and key polymorphisms in culture-amplified shed nOPV2 virus meeting predefined criteria to conduct multidose assay (≥40% paralysis in two-dose assay)

	**EES day**	**Paralysis rate 4 log**_10_**CCID**_50_**virus dose**	**Paralysis rate 5 log**_10_**CCID**_50_**virus dose**	PD_50_*log_10_ CCID_50_ (95% CI)	**Mutations in Cre5 at nucleotides 123 or 179, % in isolate**	**Mutations in Cre5 at nucleotide 181, % in isolate**	**Domain IV 459u→c**†**, % in isolate**	**Domain V SNPs in isolate**	**VP1-143 total, % in isolate**	**3D-pol-Arg38Lys, % in isolate**
M5-1-556	15	10%	50%	6·3 (5·8–6·7)	98%	..	35%	..	99%	6%
M5-1-418	20	0	40%	5·8 (5·4–6·2)	98%	5%	14%	4%; 547C→U	87%	..
M5-2-416	21	0	40%	6·0 (5·3–6·7)	97%	9%	71%	16%; 566A→G	84%	8%
M5-2-393	28	0	40%	5·5 (5·0–5·9)	99%	..	70%	..	100%	..
M5-2-351	28	40%	60%	5·3 (4·7–5·9)	100%	..	99%	97%; 556C→U	99%	97%

3D-pol=3D-polymerase. CCID_50_=50% cell culture infectious dose. Cre5=cis-acting replication element 5. EES=exploratory endpoint specimen. nOPV2=novel oral poliovirus vaccine 2. PD_50_=paralytic dose 50%. SNP=single-nucleotide polymorphism.

* Compared to nOPV2 with no estimable PD_50_value (no virulence at any dose tested in mice), and PD_50_ of 3·5 for shed Sabin-2 at day 7 after vaccination (Sabin-2 has a PD_50_ of 6·3).

† Nucleotide position 398 in Sabin-2.

Logistic regression analyses assessed the association of mutations known to increase virulence of Sabin-2 with observed rates of paralysis in the mTgmNVT. For nOPV2, mutations identified in the modified regions of shed virus as well as the unprotected secondary attenuation site, VP1-143, were assessed. Unadjusted logistic regression analyses (appendix p 12) indicate that virulence of shed mOPV2 is positively associated with higher rates of the 398u→c, 481a→g, and VP1-143 mutations. However, the aORs (table 2; appendix p 13) indicate that virulence in mice, at both inoculum doses, is driven primarily by reversion at 481 in domain V. For nOPV2 the mutations in cre5 (123 or 179) and VP1-143 appear associated with increased paralysis rates observed in mice at the 5 log_10_ CCID_50_ dose level (table 2; appendix p 13). These results are consistent with the data generated from the molecular constructs (appendix p 31).

**Table 2:** Adjusted odds ratios of mouse paralysis associated with 10% frequency difference of key mutations of shed virus in EES, estimated from a binomial logistic generalised linear regression mixed model, adjusted for mouse sex, key mutations, and with subject-specific random effects

	**4 log**_10_**CCID**_50_**virus per 5 μL inoculum**		**5 log**_10_**CCID**_50_**virus per 5 μL inoculum**	
	aOR (95% CI)	p value	aOR (95% CI)	p value
**mOPV2**				
Domain IV 398	1·31 (0·45–3·81)	0·62	1·26 (0·29–5·55)	0·76
Domain IV 481	1·47 (1·25–1·74)	<0·0001	1·45 (1·18–1·78)	0·0004
VP1-143	1·04 (0·80–1·35)	0·76	1·16 (0·72–1·86)	0·55
**nOPV2**				
Cre5 123 or 179	1·14 (0·76–1·72)	0·53	1·36 (1·14–1·62)	0·0006
Domain IV 459	1·39 (0·93–2·08)	0·11	1·06 (0·92–1·23)	0·43
VP1-143	0·94 (0·65–1·35)	0·72	1·33 (1·14–1·55)	0·0002

Data are adjusted odds ratio (95% CI) and p values. CCID_50_=50% cell culture infectious dose. Cre5=cis-acting replication element 5. EES=exploratory endpoint specimen. mOPV2=monovalent type 2 oral poliovirus vaccine. nOPV2=novel type 2 oral poliovirus vaccine.

## Discussion

The primary objective of nOPV2 development was to substantially reduce the potential for reversion to virulence observed with Sabin-2. Paralysis rates in the transgenic mouse model used in this study support the conclusion that virus amplified from stool of nOPV2 recipients are significantly less virulent than virus isolated from stool of mOPV2 recipients. We thus conclude that nOPV2 is genetically and phenotypically more stable than mOPV2.

Reversion of Sabin-2 virus occurs during replication in the gut of most vaccine recipients, but VAPP is rare. It is probable that reduced or delayed replication of the vaccine virus in the gut allows the innate and possibly adaptive immune responses to develop in most individuals, preventing development of poliomyelitis.^21^ Although it is unclear why certain vaccine recipients are susceptible to disease, the onset of paralysis in recipient VAPP is usually 7–21 days after vaccine administration. As shown in figure 2A, during this period there is a pronounced difference in neurovirulence of nOPV2 compared with shed Sabin-2 virus in the mouse model, presumably further increasing the likelihood that the induced immunity will control vaccine virus replication before neuroinvasion. In addition to the immediate risk to vaccinees, OPVs are associated with transmission to contacts and, in areas with low immunity, to communities. Although direct data on transmitted virus is not available yet, the trial results are promising, as shedding rates of nOPV2 declined more rapidly than those of mOPV2,^20^ potentially suggesting that shed nOPV2 is likely to be less transmissible.

As expected, Sabin-2 virus reverted rapidly, with shed virus showing known key genetic reversions soon after vaccination and corresponding high paralysis rates in mice once reverted, consistent with historical data for excreted Sabin-2 viruses^1^, ^2^ and molecular clones with relevant mutations (eg, 481a→g; appendix p 31). In contrast and importantly, for nOPV2, there were no reverting mutations in the main site of attenuation (domain V) analogous to the domain V 481a→g reversion in Sabin-2. the novel domain V was designed and is stabilised through exclusive use of c–g and u–a genetic pairs in stem regions. As a result, the thermostability of domain V (and thus neurovirulence) could only be increased by an improbable double mutation event that would involve a less-fit intermediate (u–a to u–g to c–g pairing), which has not been observed even after prolonged virus replication in cell-culture or humans.^10^, ^12^, ^15^ Although polymorphisms were observed in domain V, the absence of genetic variants showing such pair strengthening is consistent with the predicted genetic stability of the design of this domain.

For shed nOPV2 in five EES with paralysis rates of 40% or more in mice (table 1), the PD_50_ estimates indicate a trend towards lower values for later EES (eg, day 28 EES have the lowest PD_50_) consistent with the presence of cre5, domain IV and VP1-143 mutations in the shed virus. These mutations appear to accumulate rapidly in nOPV2 (figure 1A) perhaps in response to selective pressures given the absence of reversion in domain V. Importantly, the virus in day 28 nOPV2 EES with five fixed mutations is less virulent in mice than most of the shed mOPV2 viruses (figure 3) and is unlikely to approach the virulence of reverted Sabin-2 unless the genetically stabilised S15 domain V is replaced via a recombination event at the 5ʹ end along with a second recombination to ensure that a functional cre is incorporated into the P2 region of the genome. In conclusion, our results suggest that any significant further increase (above the observations for day 28 EES) in virulence of shed nOPV2 virus would require reversion mechanism(s) we have not observed in these clinical trials.

Although there were limitations in the M2 and M5 trials, including non-contemporaneous enrolment of participants, limitations on the number of stools that could be collected within fixed post-dose intervals, and having sufficient titre to be eligible for genetic and phenotypic evaluation, given the similarity of the infants enrolled in these trials, the data from these cohorts provide the most meaningful comparison of the neurovirulence of shed nOPV2 to shed mOPV2. Although, there is no direct way to quantitatively extrapolate to reduced risk of paralysis in humans, the available data support the increased genetic stability of domain V of nOPV2 relative to mOPV2, with significantly lower neurovirulence of shed nOPV2 virus than shed mOPV2. Additionally, the consistently lower neurovirulence of shed nOPV2 than shed mOPV2 throughout the risk period for recipient VAPP is anticipated to translate into a lower risk of VAPP. Similarly, there should be a lower likelihood of nOPV2 evolving to virulent cVDPV strains following circulation in under-immunised populations.^22^ Genetic characterisation, enhanced pharmacovigilance, and environmental surveillance have been implemented to assess these possibilities as use of this vaccine has begun in outbreak settings under the WHO emergency use listing procedure.^23^, ^24^

## Data sharing

Datasets specific to this publication are available from the corresponding author upon reasonable request and contingent on the requested uses being permitted under the informed consent received from the source clinical study, where applicable. The full genome sequence of nOPV2 candidate 1 can be found in GenBank (accession number MZ245455). Sequencing data can be accessed on the Sequence Read Archive database (accession number PRJNA786548). Algorithms for polymorphism selection are described in the methods. R codes for tables and figures specific to this publication are available from the corresponding author upon reasonable request.

## Declaration of interests

We declare no competing interests. The National Institute for Biological Standards and Control, University of California, San Francisco, claim intellectual property rights associated with the nOPV2-c1. ET works for the manufacturer of nOPV2. JS and BK are employees of Viroclinics, which is paid by PATH for the conduct of next-generation sequencing and mouse neurovirulence tests. ASB was an employee of the study funder and was involved in study design and writing of the report but had no role in data collection or analysis.
