# Supplementary material for: Genetic and phenotypic stability of poliovirus shed from infants who received novel type 2 or Sabin type 2 oral poliovirus vaccines in Panama: an analysis of two clinical trials
Source: Lancet Microbe. 2022 Dec;3(12):e912–21. doi: 10.1016/S2666-5247(22)00254-3 (PMC9712124; doi:10.1016/S2666-5247(22)00254-3)
Supplement: Supplementary appendix [file mmc1.pdf]

THE LANCET
Microbe

Supplementary appendix

This appendix formed part of the original submission and has been peer reviewed. We post it as supplied by the authors.

Supplement to: Wahid R, Mercer LD, De Leon T, et al. Genetic and phenotypic stability of poliovirus shed from infants who received novel type 2 or Sabin type 2 oral poliovirus vaccines in Panama: an analysis of two clinical trials. *Lancet Microbe* 2022; published online Nov 1. https://doi.org/10.1016/S2666-5247(22)00254-3.

Contents

1.1 Analysis Plan for Neurovirulence of Shed Virus 2
1.1.1 Introduction 2
1.1.2 Descriptive Model-based Analysis – Two-dose Assay 4
1.1.3 Descriptive Model-based Analysis – Multi-dose Assay 5
1.1.4 Comparative Model-based Analysis 6
1.2 Results for Neurovirulence of Shed Virus 7
1.3 Supplemental Figures 9
1.4 Supplemental Tables 14

1.1 Analysis Plan for Neurovirulence of Shed Virus

1.1.1 Introduction

For stool samples obtained from infants receiving Sabin mOPV2 in study M2 and those in M5 receiving high doses of the 2018 lot nOPV2 candidate 1 (nOPV2), select stool samples processed through the PCR and $CCID_{50}$ assays were be selected for use in a transgenic mouse assay to assess the potential for neurovirulence (NV) of shed virus. In order to be selected into the NV assay, stool samples were to meet the following criteria:

1. Sample was obtained within the relevant post/pre-dose interval (for post-dose-1 samples, sample was obtained following the first dose and prior to any second dose)
2. Sample was positive for type 2 virus via PCR
3. Sample was negative for types 1 and 3 virus via PCR
4. Sample was of sufficient quantity to enable amplification with minimal drift from virus in stool ($CCID_{50}$ above the predetermined cutoff of 4.0 log_{10} $CCID_{50}$/g of stool)
5. Virus obtained following amplification was determined to be positive for type 2 and negative for types 1 and 3 via PCR.
6. Amplified virus was of sufficient titer to enable an evaluation of neurovirulence (not less than 4.0 log_{10} $CCID_{50}$/5µL)
7. Sample was the last sample for a given subject meeting criteria 1-3 as measured in time from vaccination.

The selected samples were labeled Exploratory Endpoint Samples (EESs). The results of the neurovirulence assay were to be analyzed descriptively, along with a comparative evaluation of the neurovirulence of EES between nOPV2 and mOPV2.

Validity criteria for the back-titration of inoculum were established to ensure the dose of inoculum was similar across assay iterations. It was possible, however, that variation or imbalance in the actual dose level administered could observed, which would affect interpretation of results. For the multi-dose formats of the study, the actual titer was therefore to be used in the model, rather than the nominal level. Hereafter, the "multi-dose assay" refers to assays with >2 dose levels (typically targeting estimation of PD_{50}), and the "two-dose assay" refers to the specific case of infant samples tested for primary comparison of samples collected in M5 vs those collected in M2 (inoculum doses of 4.0 and 5.0 log_{10} $CCID_{50}$/5µL, with primary inference drawn from the odds ratio from the model-based estimate at 4.5 log_{10} $CCID_{50}$/5µL). The two-dose assay format was created following initial results from studies M1/M4 as well as prior results among children aged 1 to 5 years, for evaluation of the samples from infants in order to improve assay robustness. The primary comparative results from the two-dose assay was to be derived from estimation of the odds ratio of mouse paralysis between the two vaccines, averaged across mouse sex, at 4.5 log_{10} $CCID_{50}$/5µL, with corresponding p-value and two-sided 95% CIs, with the values at 4.0 log_{10} $CCID_{50}$/5µL and 5.0 log_{10} $CCID_{50}$/5µL providing key supportive results.

Samples selected and assessed for neurovirulence in this assay are summarized below.

A neurovirulence endpoint assay result (NEAR) is defined as an assay result for a given EES and dose level of inoculum such that the accompanying high- and low-dose controls, and the positive control, and the back-titration of inoculum are all within acceptable limits, per SOP. Each infant with a sample tested was expected to have 1 NEAR at both the nominal dose levels of 4.0 $\log_{10}$ $CCID_{50}$/5µl and 5.0 $\log_{10}$ $CCID_{50}$/5µl. Additional NEARs at additional dose levels arose when the multidose format of the assay was initiated for a given subject/sample.

1.1.1.1 Subsampling of EES

In addition, based on prior results indicating low mouse paralysis and limited loss of attenuating mutations via NGS for nOPV2 in the first week following vaccination, and in effort to limit the large number of mice required to arrive at a conclusive result for the comparison of nOPV2 versus mOPV2, stratified random subsampling was performed to ensure n=36 EES were evaluated in this neurovirulence assay from each vaccine. There were already 36 such samples available from mOPV2 recipients, so the subsampling needed only to be employed for nOPV2, where n=51 EES were available for assessment in the assay. Among these, there were 29, 11, 8, and 3 EES available from post-vaccination weeks 1, 2, 3, and 4, respectively. A random sample of 14 of the 29 week-1 post-vaccination samples was randomly selected to be submitted to the assay, and inverse-probability weighting was to be used in the model to account for the subsampling (i.e., to expand the results obtained for the 14 assayed week 1 post-vaccination samples back up to carry the weight of 29 when computing estimates and results regarding average neurovirulence across all EES). All inverse-probability weights were then 1, except for samples from the first post-vaccination week for nOPV2 which used weight = $w = 1/p$, with p = selection probability = 14/29.

1.1.1.2 Choice of model for NV Assay

While the raw results were to be provided as a descriptive measure, the main statistical inference was predefined to be based on a model-based estimate of the mean, using a generally common and reasonable model for a binomial variable (mouse paralysis yes/no) that:

1) Is not overly complex, to avoid difficulty in interpretation
2) Could account for imbalances in the number of male/female mice evaluated for a given EES in the event that mice were not able to be scored for causes other than paralysis (e.g., inoculation trauma)
3) Could produce a single point of inference to compare the vaccines (i.e., could facilitate comparison of the two vaccines at 4.5 $\log_{10}$ $CCID_{50}$/5µl, instead of requiring multiple comparisons at 4.0 $\log_{10}$ $CCID_{50}$/5µl, and at 5.0 $\log_{10}$ $CCID_{50}$/5µl, for male mice vs female mice, etc.)
4) Did not depart substantially from the analytical methodology for the lot-release assay upon which this modified assay was based
5) Could use the actual (rather than nominal) inoculum back-titration result

a. In this manner, if inocula tended to be generally higher-than-nominal for shed virus from one vaccine relative to the other, due to either chance or systemic differences, the model-based result could provide a fair comparison by comparing predicted paralysis at a common nominal inoculum level.

6) Could incorporate inverse-probability weighting to account for stratified subsampling of EES

7) Could be easily extended to permit estimation of the PD_{50}, for samples where that was of interest.

8) Allows a more flexible view of the factors contributing to variability in the result than sampling variation alone (extra variation induced by the processes of sample production and collection, and assay variation)

The model selected for analysis was a binomial generalized mixed-effects model, described more thoroughly, below.

1.1.2 Descriptive Model-based Analysis – Two-dose Assay

The two-dose assay is the primary means of description of the data from infant samples and comparison of data from M5 to the corresponding M2 control.

For each vaccine group, a generalized linear mixed model (GLMM) was to be fitted to the binomial count of paralyzed mice for NEARs obtained at nominal the 4.0 and 5.0 $\log_{10}$ $CCID_{50}$/5µL dose levels. This model is given by

$$logit^{-1}(p_{ij}) = \beta_0 + \beta_1 I_{[sex=F]} + \beta_2 D_{ij} + \delta_i + \gamma_j \qquad (1)$$

where

- β_0 is the overall mean log-odds of paralysis for male mice
- β_1 is the difference in mean log-odds of paralysis between mouse sex (females minus males)
- β_2 is the change in log-odds of paralysis associated with a unit increase in dose level of inoculum
- D_{ij} is the actual (not nominal) dose level of inocula used for the assay.
- $I_{[X=x]}$ is the indicator function which takes the value 1 when $X=x$, and 0 otherwise
- p_{ij} is the paralysis rate for sample (subject) i, assay run j
- $\delta_i \sim N(0, \tau^2)$ is the subject-level random effect, intended to capture overdispersion due to between-sample variability in the neurovirulence of each virus population, as well as to account for duplicate observations within subject
- $\gamma_j \sim N(0, \omega^2)$ is the assay run-level random effect, intended to capture variability in the paralysis rate owing to assay run (e.g., susceptibility of groups of mice)

This model was fit in SAS/STAT software using the PROC GLIMMIX procedure, with options, EMPIRICAL (robust variance estimation), METHOD=QUAD (approximation of the marginal log-likelihood with an adaptive Gauss-Hermite quadrature), DDFM=BETWITHIN (computation of degrees of freedom for inference), and OBSWEIGHT=w (for inverse-probability weighting).

The estimated mean paralysis rate across and per mouse sex at the nominal dose levels 4.0, 4.5, and 5.0 $\log_{10}$ $CCID_{50}$/5µL was to be computed using SAS ESTIMATE statements, including the corresponding standard errors and 95% confidence intervals.

For all models, if lack of fit was detected, simplifications were to be progressively implemented beginning with removal of random effect terms to reduce to a generalized linear model (GLM) as opposed to a GLMM.

1.1.3 Descriptive Model-based Analysis – Multi-dose Assay

For nOPV2 EES that induced 40% or more paralysis, the paralytic dose for 50% of mice (PD_{50}) was to be determined by expanding the range of inocula tested, to confirm and to better characterize the neurovirulence of such samples.

In this setting the GLMM employed was:

$$logit^{-1}(p_{ijk}) = \beta_0 + \beta_1 D_{ijk} + \beta_2 I_{[sex=F]} + \delta_i \qquad (2)$$

where

- β_0 is the overall mean log-odds of paralysis for male mice
- β_1 is the change in log-odds of paralysis associated with a unit increase in dose level of inoculum
- β_2 is the difference in mean log-odds of paralysis between mouse sex (females minus males)
- $I_{[X=x]}$ is the indicator function which takes the value 1 when X=x, and 0 otherwise
- p_{ijk} is the paralysis rate for sample i at dose level j, for replicate k
- D_{ijk} indicates the *actual* (as opposed to nominal) dose level for sample i, indexed by j, for replicate k
- $\delta_i \sim N(0, \tau^2)$ is the subject-level random effect, intended to capture overdispersion due to between-sample variability in the neurovirulence of each virus population, as well as to account for duplicate observations within subject

This model was fitted in SAS/STAT software using the PROC GLIMMIX procedure, and the same options as listed above for the two-dose assay results.

If any dose level produced ≥50% of mice paralyzed, the estimated dose level corresponding to a 50% paralysis rate (PD_{50}) was to be computed using inverse-prediction from the estimated model, omitting any variance components. For male mice, this was to be obtained by solving $logit^{-1}(0.5) = \beta_0 + \beta_1 D_{ijk}$ for D_{ijk}, yielding $PD_{50} = \frac{-\beta_0}{\beta_1}$. For female mice, this was to be

obtained by solving $logit^{-1}(0.5) = \beta_0 + \beta_1 D_{ijk} + \beta_2 I_{[sex=F]}$ yielding $PD_{50} = \frac{-(\beta_0+\beta_2)}{\beta_1}$. For sex-averaged results, this was to be obtained by solving $logit^{-1}(0.5) = \beta_0 + \beta_1 D_{ijk} + \beta_2/2$ yielding $PD_{50} = \frac{-(\beta_0+\beta_2/2)}{\beta_1}$. The delta method and an assumption of asymptotic normality was to be used to compute and present the standard error of these value with accompanying 95% confidence intervals, using Normal distribution critical values.

1.1.4 Comparative Model-based Analysis

Neurovirulence data were to be compared to the corresponding M2 control data using the two-dose assay to assess neurovirulence superiority for the high-dose 2018 lot candidate 1 infant data. To do so, the following model was to be fitted:

$$logit^{-1}(p_{hij}) = \beta_0 + \beta_1 I_{[h=1]} + \beta_2 I_{[sex=F]} + \beta_3 D_{hij} + \beta_4 I_{[h=1]} D_{hij} + \delta_i + \gamma_j \qquad (3)$$

where

- h indexes virus (h =1 = control vaccine [M2], h = 2 = candidate vaccine [M5])
- i indexes sample (subject) within levels of h
- j indexes assay run within subject
- β_0 is the overall mean log-odds of paralysis for shed virus samples for male mice, control vaccine
- β_1 is the difference in mean log-odds of paralysis between the two vaccines when $D_{hij} = 0$
- β_2 is the difference in mean log-odds of paralysis between mouse sex (females minus males)
- β_3 is the change in log-odds of paralysis associated with a unit increase in dose level of inoculum
- β_4 is the difference in slope of the regression line between the two vaccines
- D_{hij} is the actual (not nominal) dose level of inocula used for the assay.
- $I_{[X=x]}$ is the indicator function which takes the value 1 when X=x, and 0 otherwise
- p_{hij} is the paralysis rate for virus source h, sample (subject) i, assay run j
- $\delta_i \sim N(0, \tau^2)$ is the subject-level random effect for shed virus, intended to capture overdispersion due to between-sample variability in the neurovirulence of each virus population, and is assumed common among all vaccine groups within a given model fit (assumed common for candidate and control samples, within the model fit)
- $\gamma_j \sim N(0, \omega^2)$ is the assay run-level random effect, intended to capture variability in the paralysis rate owing to assay run (e.g., susceptibility of groups of mice)

This model was fitted in SAS/STAT software using the PROC GLIMMIX procedure, and the same options as listed above for the descriptive results.

The odds ratio (paralysis for candidate vaccine group relative to the control group from study M2) and its corresponding two-sided 95% CI and two-sided p-value was to be computed at the following dose levels: 4.0, 4.5, and 5.0 $\log_{10}$ $CCID_{50}$/5µL, with the primary comparison coming from the SAS LSMEANS statement at the 4.5 $\log_{10}$ $CCID_{50}$/5µL dose level, and the two-sided level 0.05 hypothesis test of odds ratio (OR; nOPV2/Sabin2) equal to one (null hypothesis) vs not equal to one (alternative hypothesis), with the OR estimate in favor of reduced paralysis for nOPV2 (OR upper confidence bound < 1) required for success. Additionally, the estimated mean paralysis rate for each virus source h, $\widehat{p_h}$, was to be obtained from the results of the SAS ESTIMATE statements at dose levels 4.0, 4.5, and 5.0 $\log_{10}$ $CCID_{50}$/5µL, with corresponding 95% confidence intervals. The estimated mean paralysis rates for each mouse sex and across sex at each dose level for each vaccine was to be similarly computed and presented.

1.2 Results for Neurovirulence of Shed Virus

Whereas primary descriptive and comparative results are described in the main body of the manuscript to which this supplement pertains, the following aims to provide more detail on results of the analysis that could be not included in the main text, particularly as it pertains to distinguishing the model-estimated results from the raw results obtained from assuming inocula were equal to the nominal dose.

Descriptive results of the 2-dose assay based on independent fits of model (1) for each vaccine are depicted in **Supplemental Figure 2**. Results indicate estimated paralysis rates at nominal doses of 4.0 and 5.0 $\log_{10}$ $CCID_{50}$/µL of 0.3% (95% CI: 0.05 – 1.8) and 2.2% (95% CI: 0.5 – 9.3) for nOPV2, respectively, and 34.9% (95% CI: 20.2 – 53.2) and 84.5% (95% CI: 72.6 – 91.8) for mOPV2, respectively. At 4.5 $\log_{10}$ $CCID_{50}$/µL, estimated paralysis rates were 0.8% (95% CI: 0.2 – 3.9) and 63.1% (95% CI: 46.3 – 77.2) for nOPV2 and mOPV2, respectively.

Fit of the joint model (3) provided an estimated odds ratio of paralysis at 4.5 $\log_{10}$ $CCID_{50}$/µL of 0.007 (95% CI: 0.002 – 0.023) for nOPV/mOPV2, indicating significantly lower mouse paralysis of nOPV2 EES compared to mOPV2 EES.

Notably, back-titrated inocula were generally higher than nominal doses (see arrows on **Supplemental Figure 2**, relative to nominal inoculum doses of 4.0 and 5.0 $\log_{10}$ $CCID_{50}$/5µL). One consequence of this is that the model-estimated paralysis levels at the nominal inocula will be lower than a descriptive summary that assumes inocula were on-target, on average, and ignores the upward-biased deviations.

The multi-dose assay was triggered for 5 nOPV EES, due to observed paralysis rates ≥40% in the two-dose assay (see **Table 1** and associated description in Results of the main text). Results are depicted in **Supplemental Figure 3,** which indicate PD_{50} values ranging from 5.3 – 6.3.

1.3 Supplemental Figures

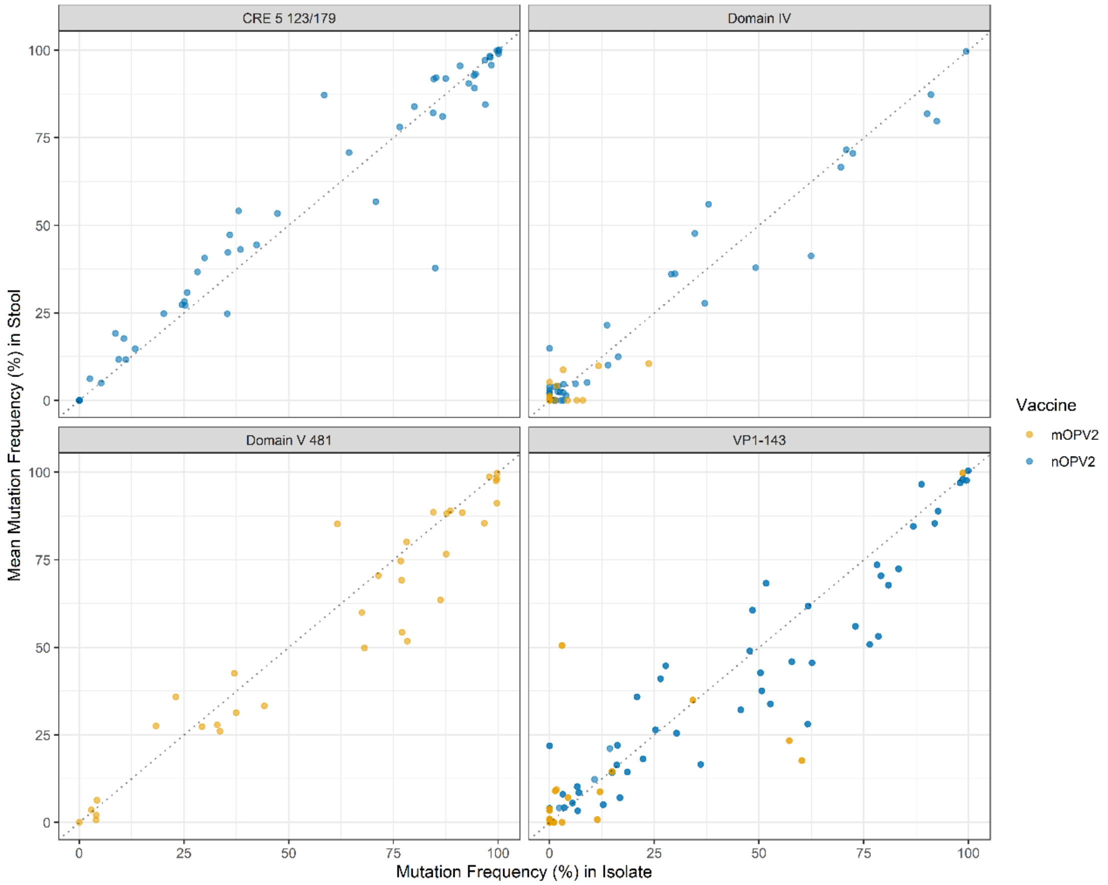

Virus	Mutation and Correlation Coefficient			
	CRE 5 123/179	Domain IV	Domain V 481	VP1-143
mOPV2	-	0.719	0.957	0.811
nOPV2	0.967	0.979	-	0.942

Supplemental Figure 1. Comparison of average frequency of selected mutations measured in EES stools and corresponding culture-amplified shed mOPV2 and nOPV2 viruses. Domain IV represents nucleotide position 398 for mOPV2 and 459 for nOPV2. VP1-143 and cre5 123/179 polymorphisms presented in aggregate for each virus. The dotted diagonal line represents the identity (1:1) line. If SNP is only present in 1 stool the other stool is assumed to be 0%. If SNP is not present in either, it is assigned 0 for plotting purposes. All reported SNPs have Q scores ≥ 30. Correlation coefficients for mutations in stool and culture-amplified shed virus are indicated per vaccine virus.

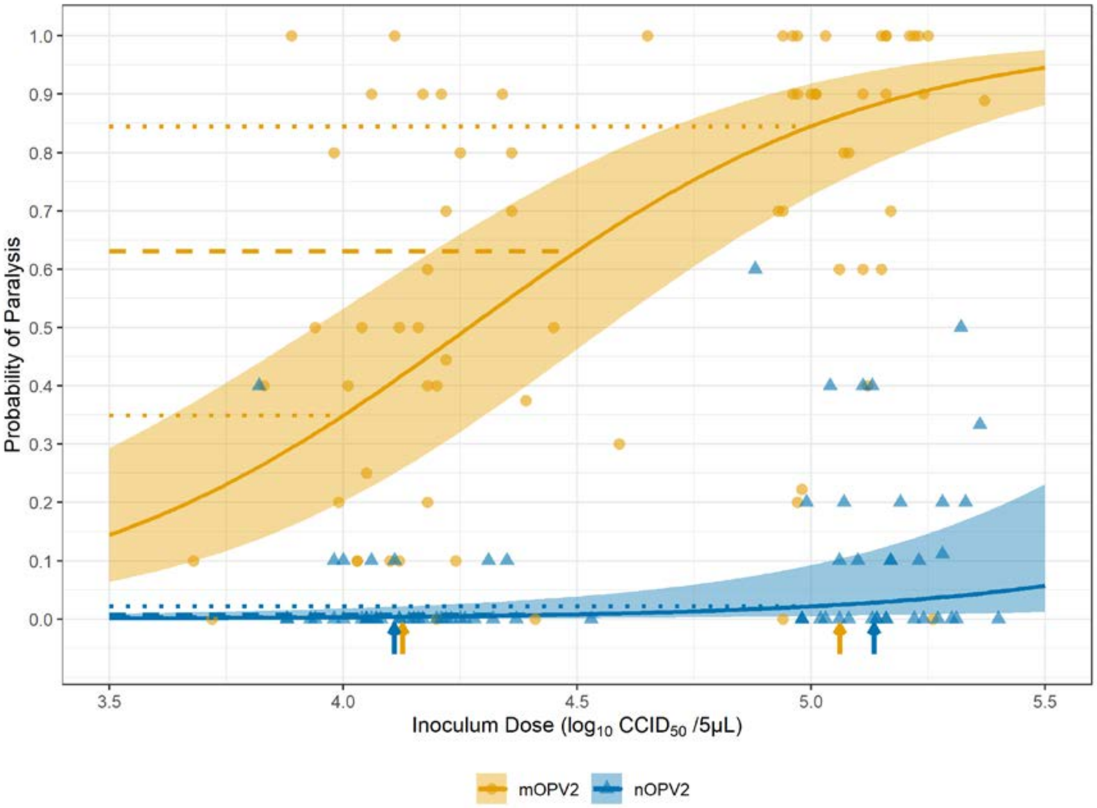

Supplemental Figure 2. Estimated paralysis rate as a function of inoculum dose, independently for nOPV2 versus mOPV2. Back-titrated inocula (means shown near nominal doses with arrows) were generally higher than the nominal doses (4.0 and 5.0 $\log_{10}$ $CCID_{50}/\mu L$). Model-based predictions at 4.0 and 5.0 $\log_{10}$ $CCID_{50}/\mu L$ are identified with dotted lines, and the model-based predictions at 4.5 $\log_{10}$ $CCID_{50}/\mu L$ with dashed lines.

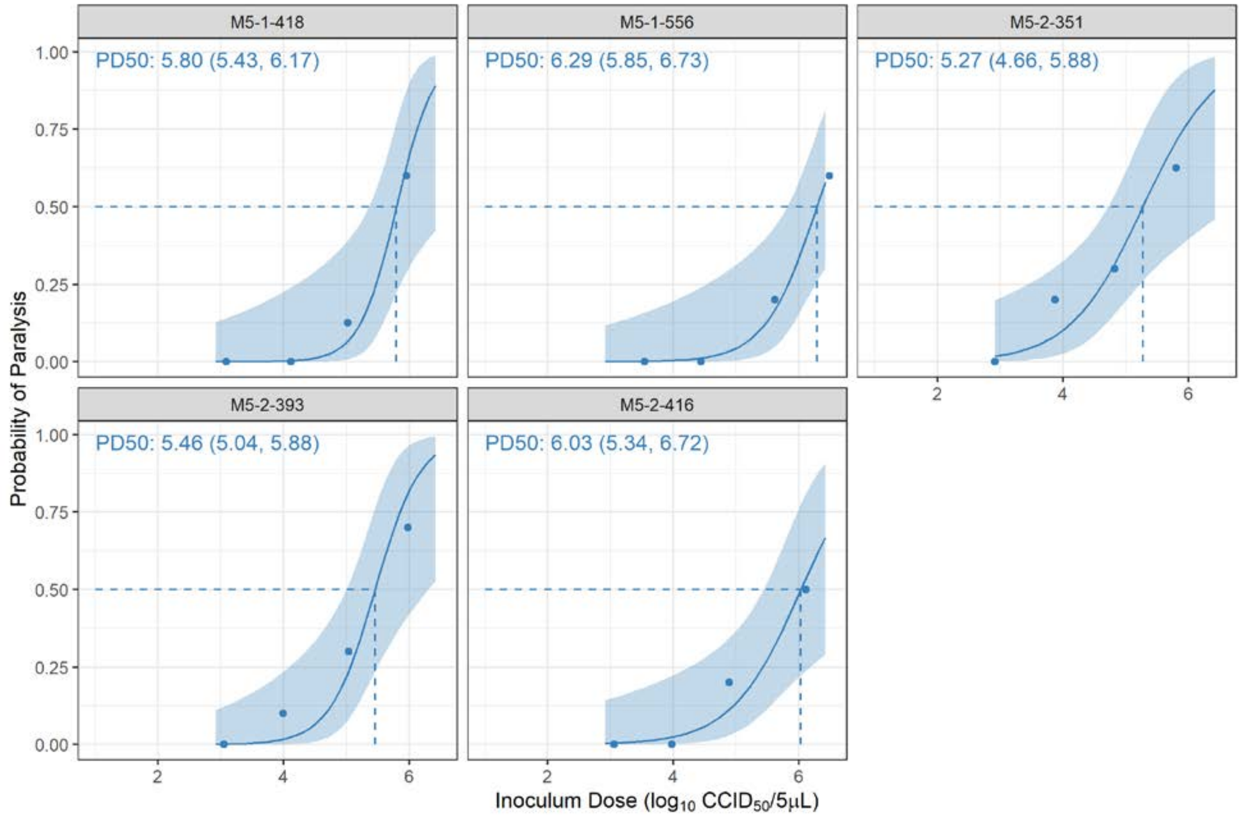

Supplemental Figure 3. Results of the multi-dose assay applied to nOPV2 EES which demonstrated ≥40% paralysis rates at a dose level in the 2-dose assay including estimated PD_{50} (dashed lines; dose level associated with 50% paralysis rates) and corresponding pointwise two-sided 95% confidence intervals.

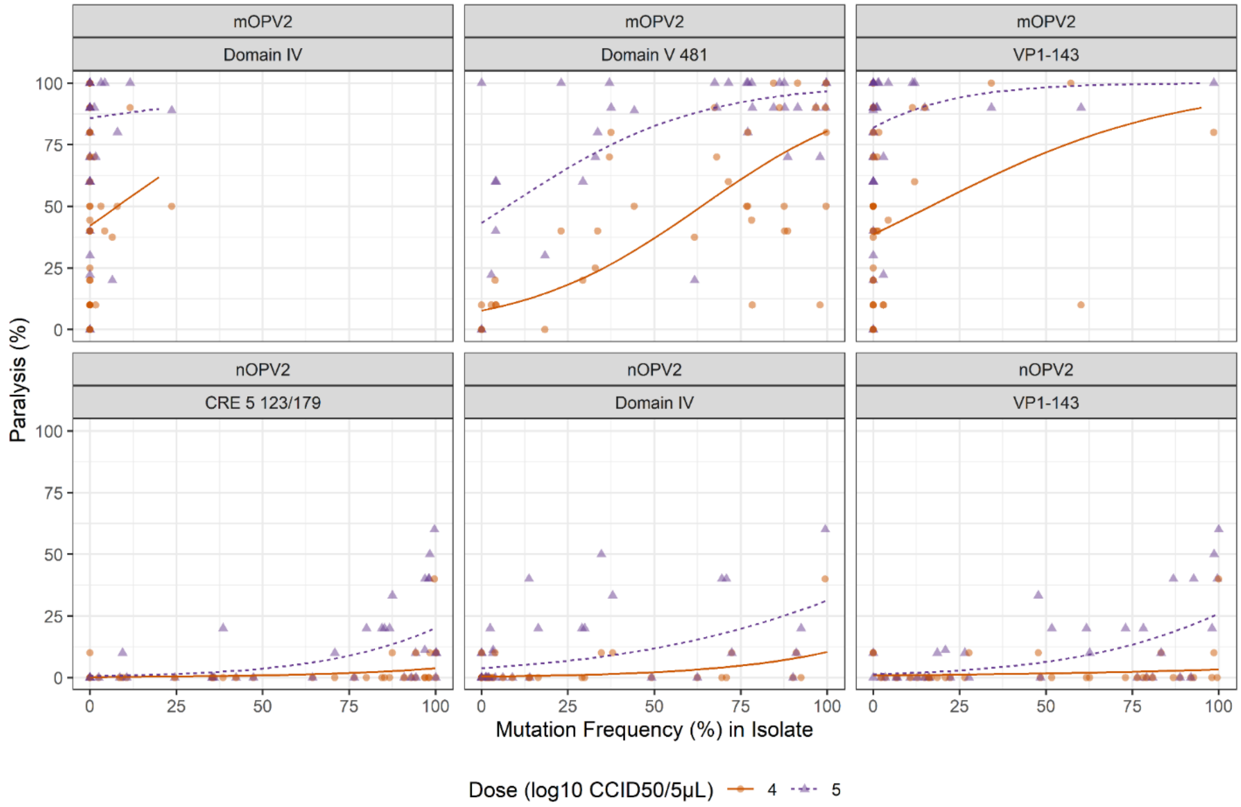

Supplemental Figure 4. Observed (points) and predicted (lines) probability of mouse paralysis as a function of mutation frequency and inoculum dose. Estimated paralysis rates derived from unadjusted univariable binomial logistic generalized linear regression model fitted to EES (36 mOPV2 and 36 nOPV2) at 10^4 $CCID_{50}$ and 10^5 $CCID_{50}$.

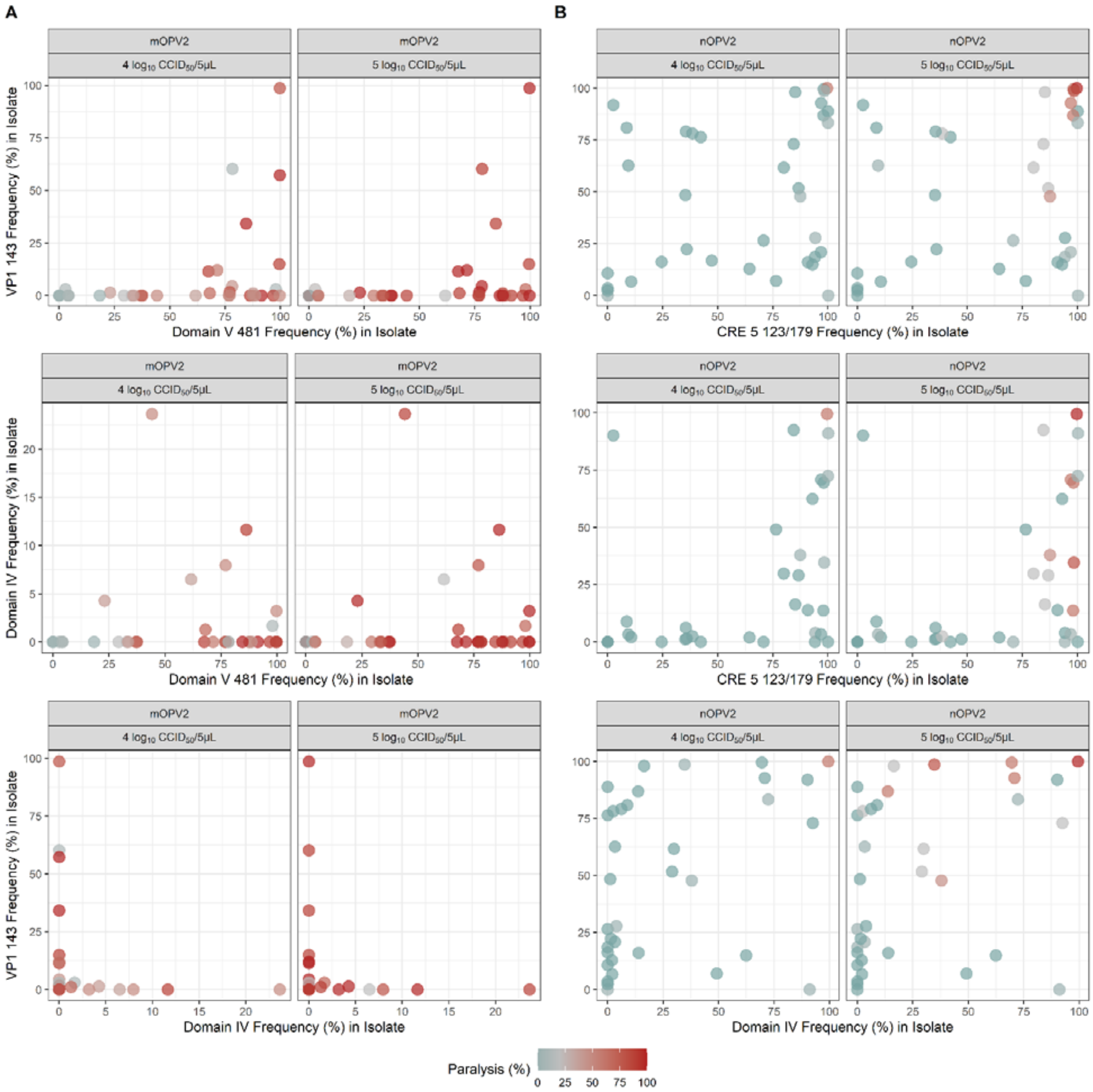

Supplemental Figure 5. Observed mouse paralysis rates (colour) by frequency of key mutations to virulence of shed virus (both x- and y-axis), by inoculum dose and vaccine. **A**. mOPV2. **B**. nOPV2.

1.4 Supplemental Tables

Supplemental Table 1. mOPV2. Frequency of polymorphisms in EES at known attenuation sites.

ID	EES Day[1]/ Variant observed[2]	Dom IV	Dom V (468-535)						VP1-143			% paralysis at 4 log_{10} $CCID_{50}$	% paralysis at 5 log_{10} $CCID_{50}$
		T398C	A481G	G491A	G493A	T500C	G505A	C517T	A2908G (I143V)	T2909C (I143T)	T2909A (I143N)		
M201066	1 (SSI)		0.01,0.03, 0.04									10	40
M201057	2 (SSI)											0	0
M201156	4 (SSI)		0.07,0.06, 0.04									10	60
M201158	4 (SSI)	0,0,0.01	0.38,0.62, 0.68				0,0.04,0					70	90
M201076	5 (SSI)		0.77,0.72, 0.77		0,0,0.01							50	100
M201100	5 (SSI)		0.87,0.89, 0.88									40	90
M201112	5 (SSI)	0,0.01,0	0.61,0.59, 0.67			0,0,0.01				0,0.02,0.11		90	100
M201120	5 (SSI)		0.27,0.29, 0.33				0,0.02,0					25	70
M201122	5 (SSI)		0.34,0.21, 0.18									0	30
M201123	5 (SSI)		0.24,0.28, 0.34		0,0,0.01	0,0,0.01						40	80
M201124	5 (SSI)		0.49,0.54, 0.78		0,0.01,0					0.17,0.16, 0.6	0.01,0.01,0	10	90
M201046	6 (SSI)		0,0.01, 0.04	0.01,0.03,0				0,0,0.02	0,0.02,0			20	60
M201068	6 (SSI)		0.29,0.34, 0.37				0,0,0.02					80	90
M201075	6 (SSI)	0.11,0.1, 0.24	0.3,0.36, 0.44	0.04,0.04, 0.04	0.01,0.01,0				0,0.01,0			50	88.89
M201084	6 (SSI)		0.46,0.39, 0.37									70	100

ID	EES Day[1]/ Variant observed[2]	Dom IV	Dom V (468-535)						VP1-143			% paralysis at 4 $\log_{10}$ $CCID_{50}$	% paralysis at 5 $\log_{10}$ $CCID_{50}$
		T398C	A481G	G491A	G493A	T500C	G505A	C517T	A2908G (I143V)	T2909C (I143T)	T2909A (I143N)		
M201069	7 (SSI)	0.12,0.07, 0.12	0.83,0.44, 0.86	0,0.33,0	0,0.01,0				0,0.02,0	0.02,0,0	0.02,0,0	90	100
M201085	7 (SSI)	0,0,0.04	0.52,0.2, 0.23	0,0,0.03		0,0.2,0.02					0,0.18, 0.01	40	100
M201127	7 (SSI)		0.79,0.75, 0.88									50	100
M201135	7 (SSI)		0.27,0.28, 0.29			0.02,0.01, 0.01						20	60
M201151	7 (SSI)		0.91,0.86, 0.91									100	90
M201080	8 (I)											0	0
M201091	8 (SSI)	0,0,0.06	0.84,0.86, 0.62									37.5	20
M201101	8 (SSI)		0.88,0.9, 0.89				0.01,0,0					40	70
M201130	8 (SSI)		0.87,0.84, 0.97						0,0.02,0			90	90
M201142	8 (SSI)		0.04,0.03, 0.03						0,0,0.03			10	22.22
M201049	9 (SSI)	0,0.03,0	0.86,0.92, 0.85	0,0,0.02	0.06,0.12, 0.06				0.31,0.3, 0.26	0.04,0.04, 0.08		100	90
M201064	9 (SSI)	0,0,0.08	0.66,0.72, 0.77	0,0.11,0.01	0.02,0.1,0	0.02,0,0				0.02,0,0		50	80
M201110	9 (I)	0.01	0.51			0.02						10	100
M201105	10 (SI)		0.54,0.77							0.09,0.02		80	100
M201126	10 (SSI)		0.7,0.71, 0.71	0,0,0.02	0.01,0,0	0.01,0, 0.01			0,0,0.02	0.1,0.07,0.1		60	100
M201044	13 (SSI)	0,0.01,0	0.78,0.82, 0.78	0,0,0.05	0.03,0,0	0,0.02,0		0.01,0.02,0	0,0.05,0	0.05,0.05, 0.04		44.44	100
M201056	14 (SSI)	0,0.08,0.02	0.98,1, 0.98						0.47,0.19, 0.03	0.22,0.14,0		10	70
M201159	14 (SSI)	0.08,0.1, 0.03	0.98,0.98,1						0.02,0.02,0	0,0.03,0		50	100

**ID**	**EES Day[1]/ Variant observed[2]**	**Dom IV**	**Dom V (468-535)**						**VP1-143**			**% paralysis at 4 $\log_{10}$ $CCID_{50}$**	**% paralysis at 5 $\log_{10}$ $CCID_{50}$**
		T398C	A481G	G491A	G493A	T500C	G505A	C517T	A2908G (I143V)	T2909C (I143T)	T2909A (I143N)		
M201061	21 (SSI)		0.99,0.83,1						0.27,0,0.3	0.1,0.09, 0.27		100	n.a.
M201145	21 (SSI)	0,0.01,0	0.99,0.97,1	0,0.01,0				0.02,0.02,0	0.1,0.07, 0.08	0.05,0.06, 0.07		90	90
M201087	28 (SSI)	0.02,0.08,0	1,1,1				0.02,0.07,0			1,1,0.99		80	100

[1]EES day shown with stool 1, stool 2 and cell culture isolate (SSI), if present; [2] Associated amino acid change indicated, if applicable. Blank cells = variant not detected in stool or isolate; Gray cells = result not available ; n.a = insufficient titre to test in mTgmNVT; ^NGS pipeline reports the variants as SNPs. Coding impact assumes changes are not in common genomes when changes in both 2908 and 2909 are seen.

Supplemental Table 2. nOPV2. Frequency of polymorphisms in modified region, cre5.

ID	EES Day[1]/Variant observed[2]	cre5 (121-181)												
		C121T	T123C	A129G	TA131T	A132G	A132T	A162G	T172G	T172A	G179A	A181G	% paralysis at 4 $\log_{10}$ $CCID_{50}$	% paralysis at 5 $\log_{10}$ $CCID_{50}$
M5-1-565	2 (SSI)													
M5-1-587	2 (SI)												0	0
M5-2-352	2 (SSI)	0.01,0.01,0										0,0,0.02	0	0
M5-2-353	2 (SSI)												10	0
M5-1-592	3 (SI)												0	0
M5-2-385	3 (SSI)		0.04,0.06,0.05											
M5-1-533	4 (SSI)		0.34,0.3, 0.28								0.16,0.27,0.2		0	0
M5-2-383	4 (SSI)	0,0.02,0	0.32,0.21,0.21			0,0,0.02					0.05,0.03,0.04			
M5-1-394	5 (SSI)		0.91,0.86,0.9	0,0,0.02							0.04,0.06,0.05	0.02,0.03,0.04		
M5-1-420	5 (SSI)		0.38,0.35,0.26								0.04,0.04,0.04			
M5-1-427	5 (SSI)	0.01,0.03,0.04	0.23,0.24,0.74								0.13,0.15,0.11			
M5-1-517	5 (SSI)	0.01,0, 0.02	0.06,0.06,0.06								0.06,0.06,0.05			
M5-1-595	5 (SSI)	0,0,0.01	0.27,0.21,0.18								0.22,0.19,0.24	0.06,0.13,0.03	0	0
M5-2-379	5 (SSI)		0.17,0.19,0.16								0.09,0.11,0.09			
M5-1-396	6 (SSI)		0.31,0.39,0.29			0.01,0, 0.04					0.07,0.08,0.06		0	0
M5-1-409	6 (SSI)		0.46,0.43,0.3								0.09,0.1,0.08			

ID	EES Day[1]/Variant observed[2]	cre5 (121-181)												
		C121T	T123C	A129G	TA131T	A132G	A132T	A162G	T172G	T172A	G179A	A181G	% paralysis at 4 $\log_{10}$ $CCID_{50}$	% paralysis at 5 $\log_{10}$ $CCID_{50}$
M5-1-528	6 (SSI)		0.88,0.88,0.8								0.04,0.04,0.05	0.01,0, 0.03		
M5-1-591	6 (SI)	0,0.01	0.63,0.26								0.24,0.32	0,0.04		
M5-1-639	6 (SSI)		0.17,0.15,0.14								0.1,0.08,0.07			
M5-2-342	6 (SSI)	0,0.01,0	0.12,0.07,0.05				0.04,0.05,0				0.05,0.05,0.08			
M5-2-346	6 (SSI)		0.26,0.24,0.25		0.07,0.01, 0						0.02,0.03,0		0	0
M5-2-357	6 (SSI)		0.13,0.17,0.07								0.04,0.04,0.02		0	0
M5-2-387	6 (SSI)	0.03,0.04,0.05	0.15,0.15,0.17								0.14,0.1,0.08	0,0,0.01		
M5-1-398	7 (SSI)		0.35,0.38,0.33				0,0.01, 0				0.11,0.03,0.05		0	20
M5-1-422	7 (SSI)	0.01,0,0	0.11,0.28,0.15								0.57,0.45,0.5		0	0
M5-1-462	7 (SSI)	0,0,0.06	0.05,0.06,0								0.05,0.08,0.09		0	10
M5-1-623	7 (SSI)		0.41,0.41,0.3								0.07,0.05,0.06		0	0
M5-2-319	7 (SSI)	0.01,0.01,0.01	0.78,0.75,0.82								0.15,0.17,0.13		0	10
M5-2-324	7 (SSI)		0.26,0.32,0.05								0.07,0.08,0.23			
M5-1-452	8 (SSI)	0.14,0.01,0	0.2,0.25, 0.3						0,0.01,0		0,0.05, 0.05		0	0
M5-2-333	8 (SSI)	0,0,0.01	0.04,0.06,0.03								0.01,0.01,0		0	0
M5-2-311	9 (SSI)		0.49,0.48,0.55						0.16,0.11,0.19		0.33,0.39,0.25	0.01,0.01,0	0	20

ID	EES Day[1]/Variant observed[2]	cre5 (121-181)												
		C121T	T123C	A129G	TA131T	A132G	A132T	A162G	T172G	T172A	G179A	A181G	% paralysis at 4 $\log_{10}$ $CCID_{50}$	% paralysis at 5 $\log_{10}$ $CCID_{50}$
M5-1-496	10 (SSI)		0.66,0.71,0.53								0.3,0.24, 0.38	0.01,0.01,0.01	0	0
M5-1-518	10 (SSI)	0,0,0.01	0.82,0.85,0.84			0.01,0.03,0.02					0.05,0.09,0.09		0	0
M5-1-579	10 (SSI)		0.88,0.92,0.83								0.09,0.05,0.14	0,0,0.02	0	11.11
M5-2-283	10 (SSI)		0.5,0.63,0.71										0	10
M5-1-372	14 (SSI)		0.85,0.78,0.72								0.1,0.12, 0.13	0,0.01,0	0	20
M5-1-527	14 (SSI)		0.8,0.78, 0.77			0.03,0, 0.25					0.11,0.15,0.11	0.02,0.02,0.02	10	33.33
M5-2-386	14 (SSI)		0.84,0.84,0.84								0.06,0.05,0.11		10	0
M5-2-420	14 (SSI)		0.98,0.98,0.9								0.02,0.02,0.1	0,0.02,0	0	0
M5-1-481	15 (SSI)	0,0,0.01	0.48,0.71,0.58								0.29,0.14,0.28	0.01,0.02,0.02	0	20
M5-1-541	15 (SSI)	0.03,0, 0.06	0.59,0.45,0.47								0.32,0.2, 0.29	0,0.01,0	0	0
M5-1-556	15 (SSI)		0.93,0.91,0.93								0.05,0.02,0.05	0.02,0,0	10	50
M5-1-418	20 (SSI)		0.96,0.98,0.98								0.02,0,0	0.09,0.04,0.05	0	40
M5-1-560	21 (SSI)		0.95,0.99,0.97								0.04,0, 0.03	0.04,0,0	10	10
M5-1-588	21 (SSI)		0.87,0.87,0.9					0,0.1,0			0.14,0.13,0.1	0.05,0.03,0	10	10
M5-2-343	21 (SSI)		0.13,0.12,0.08								0.05,0.06,0.03		0	0
M5-2-416	21 (SSI)		0.63,0.75,0.91								0.18,0.12,0.06	0.04,0.05,0.09	0	40

**ID**	**EES Day[1]/Variant observed[2]**	**cre5 (121-181)**												
		C121T	T123C	A129G	TA131T	A132G	A132T	A162G	T172G	T172A	G179A	A181G	**% paralysis at 4 log_{10} $CCID_{50}$**	**% paralysis at 5 log_{10} $CCID_{50}$**
M5-1-379	23 (SSI)		0.37,0.29,0.65	0.02,0.03,0							0.42,0.56,0.19	0.03,0,0.04	0	20
M5-2-351	28 (SSI)		1,1,1			0.01,0,0							40	60
M5-2-393	28 (SSI)	0.02,0,0	0.86,0.9,0.94							0.01,0.01,0.02	0.13,0.08,0.05		0	40

[1]EES day shown with stool 1, stool 2 and cell culture isolate (SSI), if present; [2] Associated amino acid change indicated, if applicable. Blank cells = variant not detected in stool or isolate. Gray cells = result not available.

Supplemental Table 3. nOPV2. Frequency of polymorphisms in domains IV and V.

ID	EES Day[1]/Variant observed[2]	Dom IV	S15 Dom V								% paralysis at $4log_{10}$ $CCID_{50}$	% paralysis at $5log_{10}$ $CCID_{50}$
		T459C	C547T	C550T	C556T	A566G	C569T	C578T	C582T	A588G		
M5-1-565	2 (SSI)											
M5-1-587	2 (SI)		0.05,0								0	0
M5-2-352	2 (SSI)							0.02,0.03,0.03			0	0
M5-2-353	2 (SSI)										10	0
M5-1-592	3 (SI)										0	0
M5-2-385	3 (SSI)		0,0.01,0									
M5-1-533	4 (SSI)	0.04,0,0.01	0.01,0,0								0	0
M5-2-383	4 (SSI)	0.02,0.02,0										
M5-1-394	5 (SSI)											
M5-1-420	5 (SSI)											
M5-1-427	5 (SSI)	0.02,0.02,0										
M5-1-517	5 (SSI)											
M5-1-595	5 (SSI)										0	0
M5-2-379	5 (SSI)	0.02,0.02,0.03										
M5-1-396	6 (SSI)	0.03,0.07,0.06	0.01,0.02,0.03								0	0
M5-1-409	6 (SSI)	0.02,0,0										
M5-1-528	6 (SSI)	0,0,0.01	0.03,0.03,0.02			0,0,0.01						
M5-1-591	6 (SI)	0,0.03	0,0.01			0,0.02						
M5-1-639	6 (SSI)	0.27,0.29,0.37	0.04,0,0.01			0.02,0.02,0.01						
M5-2-342	6 (SSI)				0,0.04,0		0.04,0.03,0.05					
M5-2-346	6 (SSI)	0.05,0.03,0			0,0.01,0						0	0

ID	EES Day[1]/Variant observed[2]	Dom IV	S15 Dom V								% paralysis at 4$\log_{10}$ $CCID_{50}$	% paralysis at 5$\log_{10}$ $CCID_{50}$
		T459C	C547T	C550T	C556T	A566G	C569T	C578T	C582T	A588G		
M5-2-357	6 (SSI)	0.06,0.05,0.09									0	0
M5-2-387	6 (SSI)											
M5-1-398	7 (SSI)	0.02,0.03,0.02									0	20
M5-1-422	7 (SSI)	0.03,0.02,0.02				0.01,0.01,0.01					0	0
M5-1-462	7 (SSI)	0.01*,0,0.03									0	10
M5-1-623	7 (SSI)	0,0,0.01	0,0,0.02								0	0
M5-2-319	7 (SSI)										0	10
M5-2-324	7 (SSI)	0.12,0.18,0				0,0,0.01						
M5-1-452	8 (SSI)	0,0,0.01				0.11,0,0					0	0
M5-2-333	8 (SSI)	0.85,0.79,0.9									0	0
M5-2-311	9 (SSI)	0.34,0.38,0.3		0.02,0.01,0.01		0.03,0.03,0					0	20
M5-1-496	10 (SSI)	0.11,0.09,0.14			0.01,0,0						0	0
M5-1-518	10 (SSI)	0.44,0.39,0.62	0.02,0.04,0.1							0.02,0.05,0.03	0	0
M5-1-579	10 (SSI)	0.05,0.04,0.03									0	11.11
M5-2-283	10 (SSI)				0,0,0.03						0	10
M5-1-372	14 (SSI)	0.13,0.11,0.16		0,0.01,0							0	20
M5-1-527	14 (SSI)	0.57,0.55,0.38	0.02,0.01,0.01	0.02,0.01,0.02	0,0,0.02						10	33.33
M5-2-386	14 (SSI)	0.01,0.01,0.04									10	0
M5-2-420	14 (SSI)	0.02,0.03,0									0	0
M5-1-481	15 (SSI)	0.47,0.25,0.29									0	20
M5-1-541	15 (SSI)	0.42,0.34,0.49									0	0
M5-1-556	15 (SSI)	0.47,0.48,0.35				0.02,0,0.01*					10	50
M5-1-418	20 (SSI)	0.29,0.14,0.14	0.12,0.13,0.04								0	40

ID	EES Day[1]/Variant observed[2]	Dom IV	S15 Dom V								% paralysis at 4log$_{10}$ CCID$_{50}$	% paralysis at 5log$_{10}$ CCID$_{50}$
		T459C	C547T	C550T	C556T	A566G	C569T	C578T	C582T	A588G		
M5-1-560	21 (SSI)	0.75,0.66,0.72	0.01,0.03,0.03		0.02,0.02,0.02	0.03,0.01,0			0.01,0.02,0.03		10	10
M5-1-588	21 (SSI)	0.87,0.87,0.91			0,0.01,0	0,0.01,0.01					10	10
M5-2-343	21 (SSI)	0.04,0.04,0.02		0.02,0,0							0	0
M5-2-416	21 (SSI)	0.71,0.72,0.71	0.01,0,0		0.02,0.02,0	0.06,0.07,0.16	0.02,0.02,0				0	40
M5-1-379	23 (SSI)	0.8,0.79,0.92				0,0,0.03	0.3,0.25,0.11		0,0,0.02		0	20
M5-2-351	28 (SSI)	1,1,0.99			0.81,0.85,0.97						40	60
M5-2-393	28 (SSI)	0.71,0.62,0.7									0	40

[1]EES day shown with stool 1, stool 2 and cell culture isolate (SSI), if present; [2] Associated amino acid change indicated, if applicable. Blank cells = variant not detected in stool or isolate; *Q<30 for variant in this replicate. Gray cells = result not available.

Supplemental Table 4. nOPV2. Frequency of polymorphisms in attenuating site, VP1-143.

ID	EES Day[1]/Variant observed[2]	VP1-143 (2969-2971)^					
		A2969G (I143V)	A2969T (I143F)	T2970C (I143T)	T2970A (I143N)	% paralysis at 4 log_{10} $CCID_{50}$	% paralysis at 5 log_{10} $CCID_{50}$
M5-1-565	2 (SSI)			0.08,0.08,0.03			
M5-1-587	2 (SI)	0.01,0		0.03,0.02		0	0
M5-2-352	2 (SSI)			0.04,0.04,0.04		0	0
M5-2-353	2 (SSI)			0.04,0.04,0		10	0
M5-1-592	3 (SI)	0,0.01		0.12,0.09		0	0
M5-2-385	3 (SSI)	0.05,0.05,0.02		0.04,0.06,0.05			
M5-1-533	4 (SSI)	0,0.01,0.01		0.07,0.05,0.15		0	0
M5-2-383	4 (SSI)	0.01,0.02,0.03		0.37,0.34,0.48			
M5-1-394	5 (SSI)			0.06,0.05,0.05			
M5-1-420	5 (SSI)			0.33,0.35,0.53			
M5-1-427	5 (SSI)			0.22,0.21,0	0.01,0,0		
M5-1-517	5 (SSI)	0.02,0.02,0.02		0.23,0.24,0.28			
M5-1-595	5 (SSI)			0.51,0.5,0.76		0	0
M5-2-379	5 (SSI)	0.02,0.02,0.03		0.43,0.38,0.48			
M5-1-396	6 (SSI)	0,0,0.01		0.72,0.69,0.78		0	0
M5-1-409	6 (SSI)	0.14,0.12,0.11		0.13,0.13,0.14			
M5-1-528	6 (SSI)			0.29,0.28,0.62			
M5-1-591	6 (SI)			0.21,0.14			
M5-1-639	6 (SSI)	0.06,0.06,0.05		0.25,0.28,0.39			
M5-2-342	6 (SSI)			0.54,0.52,0.78			
M5-2-346	6 (SSI)	0.03,0.04,0.1		0.16,0.21,0.06		0	0
M5-2-357	6 (SSI)	0.03,0.03,0.02		0.66,0.64,0.79		0	0
M5-2-387	6 (SSI)	0,0,0.02		0.47,0.45,0.56			
M5-1-398	7 (SSI)	0.01,0,0		0.72,0.74,0.78		0	20

ID	EES Day[1]/Variant observed[2]	VP1-143 (2969-2971)^					
		A2969G (I143V)	A2969T (I143F)	T2970C (I143T)	T2970A (I143N)	**% paralysis at 4 $\log_{10}$ $CCID_{50}$**	**% paralysis at 5 $\log_{10}$ $CCID_{50}$**
M5-1-422	7 (SSI)			0.05,0.05,0.13		0	0
M5-1-462	7 (SSI)			0.49,0.41,0.63		0	10
M5-1-623	7 (SSI)			0.19,0.17,0.22		0	0
M5-2-319	7 (SSI)	0.06,0.06,0.05		0.08,0.08,0.14		0	10
M5-2-324	7 (SSI)	0,0.01,0		0.14,0.18,0.36			
M5-1-452	8 (SSI)	0,0.02,0.02	0.2,0.08,0.21	0.55,0.37,0.26		0	0
M5-2-333	8 (SSI)			0.85,0.86,0.92		0	0
M5-2-311	9 (SSI)			0.64,0.6,0.62		0	20
M5-1-496	10 (SSI)			0.17,0.16,0.16		0	0
M5-1-518	10 (SSI)			0.13,0.12,0.15	0.03,0,0	0	0
M5-1-579	10 (SSI)			0.35,0.37,0.21		0	11.11
M5-2-283	10 (SSI)	0.03,0.03,0.01		0.39,0.38,0.25		0	10
M5-1-372	14 (SSI)			0.98,0.96,0.98		0	20
M5-1-527	14 (SSI)			0.49,0.49,0.48		10	33.33
M5-2-386	14 (SSI)	0.25,0.22,0.05		0.2,0.23,0.23		10	0
M5-2-420	14 (SSI)			0.96,0.97,0.89		0	0
M5-1-481	15 (SSI)	0.02,0,0.03		0.71,0.63,0.48		0	20
M5-1-541	15 (SSI)			0.07,0.1,0.07		0	0
M5-1-556	15 (SSI)			0.98,0.98,0.99		10	50
M5-1-418	20 (SSI)			0.82,0.87,0.87		0	40
M5-1-560	21 (SSI)	0,0.03,0.02		0.74,0.67,0.81		10	10
M5-1-588	21 (SSI)					10	10
M5-2-343	21 (SSI)	0.02,0.01,0		0,0,0.01	0.02,0.01,0.04	0	0
M5-2-416	21 (SSI)	0,0.02,0.1		0.88,0.88,0.83		0	40
M5-1-379	23 (SSI)	0.02,0.01,0		0.57,0.51,0.73		0	20
M5-2-351	28 (SSI)	0.87,0.88,0.97		0.14,0.12,0.02		40	60
M5-2-393	28 (SSI)			0.98,0.98,1		0	40

ID	EES Day[1]/Variant observed[2]	VP1-143 (2969-2971)^					
		A2969G (I143V)	A2969T (I143F)	T2970C (I143T)	T2970A (I143N)	**% paralysis at 4 $\log_{10}$ $CCID_{50}$**	**% paralysis at 5 $\log_{10}$ $CCID_{50}$**
[1]EES day shown with stool 1, stool 2 and cell culture isolate (SSI), if present; [2] Associated amino acid change indicated, if applicable. Blank cells = variant not detected in stool or isolate; Blank cells = variant not detected in stool or isolate. Gray cells = result not available. ^NGS pipeline reports the variants as SNPs. Coding impact assumes changes are not in common genomes when changes in both 2969 and 2970 are seen.							

Supplemental Table 5. nOPV2. Frequency of polymorphisms in modified cre knockout region and 3D polymerase.

ID	EES Day[1]/ Variant observed[2]	2C cre KO (4508-4560)										3D-pol-38 (6158-6160)		3D-pol-53 (6203-6205)	% paralysis	
		T4510C	T4513C	C4519T	T4525C	T4540C	C4543T	A4549G	T4555C	G4558A	T4559C	G6159A (R38K)	A6160G	T6205C	4 log	5 log
M5-1-565	2 (SSI)															
M5-1-587	2 (SI)														0	0
M5-2-352	2 (SSI)														0	0
M5-2-353	2 (SSI)														10	0
M5-1-592	3 (SI)					0,0.04									0	0
M5-2-385	3 (SSI)															
M5-1-533	4 (SSI)	0.04,0.04, 0.03													0	0
M5-2-383	4 (SSI)															
M5-1-394	5 (SSI)	0,0,0.09														
M5-1-420	5 (SSI)											0,0.01,0				
M5-1-427	5 (SSI)															
M5-1-517	5 (SSI)															
M5-1-595	5 (SSI)														0	0
M5-2-379	5 (SSI)															
M5-1-396	6 (SSI)														0	0
M5-1-409	6 (SSI)															
M5-1-528	6 (SSI)	0.2,0.21, 0.13														
M5-1-591	6 (SI)															
M5-1-639	6 (SSI)															
M5-2-342	6 (SSI)															
M5-2-346	6 (SSI)														0	0
M5-2-357	6 (SSI)														0	0

ID	EES Day[1]/ Variant observed[2]	2C cre KO (4508-4560)										3D-pol-38 (6158-6160)		3D-pol-53 (6203-6205)	% paralysis	
		T4510C	T4513C	C4519T	T4525C	T4540C	C4543T	A4549G	T4555C	G4558A	T4559C	G6159A (R38K)	A6160G	T6205C	4 log	5 log
M5-2-387	6 (SSI)															
M5-1-398	7 (SSI)														0	20
M5-1-422	7 (SSI)		0.01,0.01,0.03												0	0
M5-1-462	7 (SSI)														0	10
M5-1-623	7 (SSI)														0	0
M5-2-319	7 (SSI)														0	10
M5-2-324	7 (SSI)	0,0.01,0														
M5-1-452	8 (SSI)											0,0.01,0			0	0
M5-2-333	8 (SSI)														0	0
M5-2-311	9 (SSI)							0,0,0.01				0.09,0.07,0.04			0	20
M5-1-496	10 (SSI)							0,0,0.02							0	0
M5-1-518	10 (SSI)														0	0
M5-1-579	10 (SSI)						0,0,0.06					0.01,0.02,0			0	11.11
M5-2-283	10 (SSI)														0	10
M5-1-372	14 (SSI)														0	20
M5-1-527	14 (SSI)							0.02,0.02,0.04					0.01,0.03,0		10	33.33
M5-2-386	14 (SSI)														10	0
M5-2-420	14 (SSI)														0	0
M5-1-481	15 (SSI)			0.03,0,0.01								0.02,0.07,0.02		0.43,0.42,0.29	0	20
M5-1-541	15 (SSI)						0.01,0,0		0.08,0.04,0.06	0.01,0.01,0					0	0
M5-1-556	15 (SSI)				0,0.01,0							0.02,0.04,0.06			10	50

ID	EES Day[1]/ Variant observed[2]	2C cre KO (4508-4560)										3D-pol-38 (6158-6160)		3D-pol-53 (6203-6205)	% paralysis	
		T4510C	T4513C	C4519T	T4525C	T4540C	C4543T	A4549G	T4555C	G4558A	T4559C	G6159A (R38K)	A6160G	T6205C	4 log	5 log
M5-1-418	20 (SSI)	0,0,0.02					0,0.02,0								0	40
M5-1-560	21 (SSI)					0,0,0.04						0,0,0.02			10	10
M5-1-588	21 (SSI)	0.01,0.02, 0				0.03,0.01, 0	0.01,0, 0.02					0.01,0.01, 0.02			10	10
M5-2-343	21 (SSI)										0.01,0.01, 0.02				0	0
M5-2-416	21 (SSI)											0.03,0.06, 0.08			0	40
M5-1-379	23 (SSI)									0.01,0,0					0	20
M5-2-351	28 (SSI)											0.82,0.87, 0.97			40	60
M5-2-393	28 (SSI)				0.01,0.01, 0										0	40

[1]EES day shown with stool 1, stool 2 and cell culture isolate (SSI), if present; [2] Associated amino acid change indicated, if applicable. Blank cells = variant not detected in stool or isolate. Gray cells = result not available.

Supplemental Table 6. M2 (mOPV2) and M5 (nOPV2) Infant EES evaluated in the mTgmNVT subsampled with simple random sampling within strata defined by post-vaccination week

	Sabin mOPV2		nOPV2	
EES Interval (Study Days)	N EES Available	N EES Tested	N EES Available	N EES tested
D1 – D7	20	20	29	14
D8 – D14	13	13	11	11
D15 – D21	2	2	8	8
D22 – D28	1	1	3	3

Supplemental Table 7. Paralytic dose 50% (PD_{50}) for in vitro molecular constructs containing key mutations identified in the EES.

Construct	PD_{50} [1]log $CCID_{50}$
nOPV2	>8.4 (0/8)[2]
nOPV2/U123C	>6.5 (2/8)[2]
nOPV2/I1143V	7.8
nOPV2/U123C/I1143V	5.4
nOPV2/U123C/U459C/I1143V	4.3
S2/481G	1.8

[1] Virulence of viruses was determined in Tg66 mice using intraspinal inoculation
[2] Proportion of mice paralysed at highest dose
